# A Placenta Derived C-Terminal Fragment of β-Hemoglobin With Combined Antibacterial and Antiviral Activity

**DOI:** 10.3389/fmicb.2020.00508

**Published:** 2020-04-06

**Authors:** Rüdiger Groß, Richard Bauer, Franziska Krüger, Elke Rücker-Braun, Lia-Raluca Olari, Ludger Ständker, Nico Preising, Armando A. Rodríguez, Carina Conzelmann, Fabian Gerbl, Daniel Sauter, Frank Kirchhoff, Benjamin Hagemann, Jasmina Gačanin, Tanja Weil, Yasser B. Ruiz-Blanco, Elsa Sanchez-Garcia, Wolf-Georg Forssmann, Annette Mankertz, Sabine Santibanez, Steffen Stenger, Paul Walther, Sebastian Wiese, Barbara Spellerberg, Jan Münch

**Affiliations:** ^1^Institute of Molecular Virology, Ulm University Medical Center, Ulm, Germany; ^2^Institute of Medical Microbiology and Hygiene, Ulm University Medical Center, Ulm, Germany; ^3^Department of Medicine I, University Hospital of Dresden, Dresden, Germany; ^4^Core Facility Functional Peptidomics, Ulm University Medical Center, Ulm, Germany; ^5^Core Unit of Mass Spectrometry and Proteomics, Ulm University, Ulm, Germany; ^6^Max Planck Institute for Polymer Research, Mainz, Germany; ^7^Institute of Inorganic Chemistry I, University of Ulm, Ulm, Germany; ^8^Computational Biochemistry, Faculty of Biology, University of Duisburg-Essen, Essen, Germany; ^9^Pharis Biotec GmbH, Hanover, Germany; ^10^WHO Measles/Rubella European RRL and NRC Measles, Mumps, Rubella, Robert Koch-Institute, Berlin, Germany; ^11^Central Facility for Electron Microscopy, Ulm University, Ulm, Germany

**Keywords:** placenta, AMP, hemoglobin fragment, proteolytic generation, antiviral

## Abstract

The placenta acts as physical and immunological barrier against the transmission of viruses and bacteria from mother to fetus. However, the specific mechanisms by which the placenta protects the developing fetus from viral and bacterial pathogens are poorly understood. To identify placental peptides and small proteins protecting from viral and bacterial infections, we generated a peptide library from 10 kg placenta by chromatographic means. Screening the resulting 250 fractions against Herpes-Simplex-Virus 2 (HSV-2), which is rarely transmitted through the placenta, in a cell-based system identified two adjacent fractions with significant antiviral activity. Further rounds of chromatographic purification and anti-HSV-2 testing allowed to purify the bioactive peptide. Mass spectrometry revealed the presence of a 36-mer derived from the C-terminal region of the hemoglobin β subunit. The purified and corresponding chemically synthesized peptide, termed HBB(112–147), inhibited HSV-2 infection in a dose-dependent manner, with a mean IC_50_ in the median μg/ml range. Full-length hemoglobin tetramer had no antiviral activity. HBB(112–147) did not impair infectivity by direct targeting of the virions but prevented HSV-2 infection at the cell entry level. The peptide was inactive against Human Immunodeficiency Virus Type 1, Rubella and Zika virus infection, suggesting a specific anti-HSV-2 mechanism. Notably, HBB(112–147) has previously been identified as broad-spectrum antibacterial agent. It is abundant in placenta, reaching concentrations between 280 and 740 μg/ml, that are well sufficient to inhibit HSV-2 and prototype Gram-positive and -negative bacteria. We here additionally show, that HBB(112–147) also acts potently against *Pseudomonas aeruginosa* strains (including a multi-drug resistant strain) in a dose dependent manner, while full-length hemoglobin is inactive. Interestingly, the antibacterial activity of HBB(112–147) was increased under acidic conditions, a hallmark of infection and inflammatory conditions. Indeed, we found that HBB(112–147) is released from the hemoglobin precursor by Cathepsin D and Napsin A, acidic proteases highly expressed in placental and other tissues. We propose that upon viral or bacterial infection, the abundant hemoglobin precursor is proteolytically processed to release HBB(112–147), a broadly active antimicrobial innate immune defense peptide.

## Introduction

The placenta is a fetal organ tightly interacting with maternal blood vessels to nourish and protect the fetus (Robbins and Bakardjiev, 2012). Placental infections by pathogens are major causes of disease and represent a substantial source of human morbidity and mortality. The placenta is composed of villi that float in maternal blood and organize the exchange of substances between the mother and fetus. At this interface bacteria and viruses may enter the placenta and be transmitted to the fetus. Thus, the placenta is an important transmission site for viral and bacterial infections, and therefore it is conceivable that this organ evolved antiviral and antibacterial innate immune defense mechanisms that prevent or restrict diaplacental pathogen transmission. Indeed, only relatively few pathogens are capable of placental and fetal infections in humans (Robbins and Bakardjiev, 2012). For example, most viral dia-placental transmissions are caused by Cytomegalovirus, Lymphocytic choriomeningitis virus, Parvovirus B19, Rubella Virus and Varicella zoster virus (Robbins and Bakardjiev, 2012), but generally the frequencies are low. More recently, it has also been shown that Zika virus (ZIKV) may cross the placenta and infect the fetus which may result in severe developmental defects of the fetus (Zanluca et al., 2018). However, for the majority of viral pathogens the placenta represents an impenetrable barrier (Robbins and Bakardjiev, 2012). For example, Herpes simplex viruses (HSV) -1 and -2 have infected billions of people. In the US, neonatal infection with HSV occurs in 1 out of every 3200–10,000 live births and is associated with serious morbidity and mortality, and leaves many survivors with permanent sequelae (Brown et al., 2003; Mahnert et al., 2007; Roberts, 2009; Flagg and Weinstock, 2011). Interestingly, most congenital HSV infections are transmitted during delivery whereas reports on transplacental transmission and fetal infection are extremely rare (Koi et al., 2002), despite a relatively high prevalence of up to 9% of HSV-2 DNA in placental tissue samples (Finger-Jardim et al., 2014). Likewise, as part of the innate immune system, the placenta plays an important role in prevention of bacterial infection during pregnancy (Mor and Kwon, 2015). Trophoblast cells carry toll-like receptors and thus are able to recognize bacteria via pattern recognition receptors to elicit an immune response (Abrahams et al., 2004). Failure to prevent bacterial infection during pregnancy may result in the termination of pregnancy and preterm delivery. While bacterial infections have clearly been implicated as one of the most important reason for preterm deliveries, the specific bacterial species that are responsible remain elusive. Nevertheless, this situation underlines the importance of an intact placental barrier. The potent barrier function that prevents dia-placental transmission of pathogens is likely due to a lack of intercellular junctions of the syncytiotrophoblast comprising most of the maternofetal interface and an environment rich in innate immune defense molecules (Robbins and Bakardjiev, 2012).

One part of the innate immune response consists of antimicrobial peptides (AMPs) that are constantly expressed or released upon TLR stimulation (Hertz et al., 2003). We have previously shown that peptide libraries generated from human body fluids are an excellent source for the discovery of novel AMPs with antibacterial and antiviral activity (Münch et al., 2014; Bosso et al., 2018). Such peptide libraries are generated from pooled body fluids by chromatographic means and typically consist of 100–400 peptide fractions that contain all peptides and small proteins of the respective source material in a lyophilized and concentrated form. Screening these libraries for antibacterial fractions and subsequent purification of the bioactive peptides allowed to identify, e.g., the first human β-defensin hBD-1 (Bensch et al., 1995), antibacterial peptides LEAP-1 and 2 (Krause et al., 2000; Krause et al., 2003), or Casein k(63–117) (Liepke et al., 2001). Using cell-based viral infection assays, we also discovered endogenous modulators of HIV-1 infection, such as a novel chemokine ligand of CCR5 that blocks HIV infection (Detheux et al., 2000; Münch et al., 2002), and a fragment of antitrypsin that inhibits HIV entry and has been successfully tested in a phase I/II clinical trial (Münch et al., 2007b; Forssmann et al., 2010). More recently, it led to the discovery of an endogenous CXCR4 antagonist that blocks CXCR4-tropic HIV infection (Zirafi et al., 2015). Moreover, screening a semen derived peptide library identified amyloid-forming peptides as enhancers of HIV-1 infection (Münch et al., 2007a; Arnold et al., 2012; Röcker et al., 2018) and antibacterial agents (Easterhoff et al., 2013). Thus, the concept of isolating novel AMPs from body fluids resulted in the identification of peptides that inhibited bacterial and viral infections by novel and unexpected mechanisms. Here, we sought to adapt this technology and generated a peptide library from human placenta to identify endogenous inhibitors that may restrict HSV-2 infection and bacterial pathogens.

## Materials and Methods

### Generation of Peptide Library From Placenta

Ten kilograms of human placenta were obtained from healthy individuals in a maternity ward of a local hospital and processed immediately after delivery. This study was approved by the Ethics Committee of Ulm University (file number 90/17). All subjects gave written informed consent in accordance with the Declaration of Helsinki. First, the organs were homogenized and peptides and small proteins extracted by an ice-cold acetic acid extraction procedure. Thereafter, the obtained peptides and proteins were separated by an ultrafiltration step (cut-off: 50 kDa). The resulting peptides were then separated in a first dimension by means of their isoelectric point using cation exchange chromatography resulting in pH pool fractions 1–6. Each pH pool was then further separated in a second dimension by peptide hydrophobicity using reversed phase chromatography resulting in a total of 250 fractions as described in detail before (Liepke et al., 2001, 2003).

### Screening the Placenta Library for Inhibitors of HSV-2 Infection

ELVIS^TM^ cells are genetically engineered baby hamster kidney cells that encode a lacZ gene, which is expressed upon infection via the viral transactivator ICP10 (Enzyme-Linked Virus-Inducible System – ELVIS^TM^) (Proffitt and Schindler, 1995). 5,000 ELVIS cells were seeded into 96-well plates and mixed with 10 μl of the reconstituted fractions of the placenta library. After 10 min, cells were infected with a clinical HSV-2 isolate (Krüger et al., 2019) and infection was determined 2 days later by detecting the β-galactosidase activity in cellular lysates using the Gal-Screen β-Galactosidase Reporter Gene Assay System for Mammalian Cells (Thermo Fisher Scientific) and the Orion II microplate luminometer (Berthold, Bad Wildbad, Germany). All values represent reporter gene activities derived from triplicates minus background activities derived from uninfected cells. Triplicates are expressed as mean ± standard error derived from two independent experiments. All experiments with infectious viruses were performed in a BSL3^∗∗^ laboratory in accordance with biosafety guidelines by Ulm University Hospital.

### Mass Spectrometry

MALDI TOF MS (Matrix-assisted laser desorption/ionization time of flight mass spectrometry) was carried out using a LaserTec RBT II (PerSeptive Biosystems, Framingham, MA, United States) as described in detail earlier (Ständker et al., 2010). The instrument is equipped with a 1.2 m flight tube and a 337 nm nitrogen laser. Positive ions are accelerated at 30 kV and laser shots are automatically accumulated per sample. Alpha-Cyano-4-hydroxy-cinnamic acid (CHC, Sigma–Aldrich, Deisenhofen, Germany) was used as matrix. Accuracy of mass measurement was 0.5%. Electrospray MS was carried out on an LCQ ion trap mass spectrometer (Finnigan/Thermo Fisher Scientific, Bonn, Germany) with an electrospray interface (ESI–MS) as described previously (John et al., 2005).

### Peptide Synthesis

Native HBB(112–147) was isolated in large amounts from human placenta. Synthetic HBB(112–147) was produced using conventional solid phase Fmoc chemistry and a Liberty blue microwave peptide synthesizer (CEM corporation, Matthews, NC, United States). The synthetic peptide showed identical chromatographic and mass spectrometric properties compared to the native peptide (purity > 95%).

### Effect of HBB(112–147) on HSV-1 and HSV-2 Infection

**FIGURE 1:**
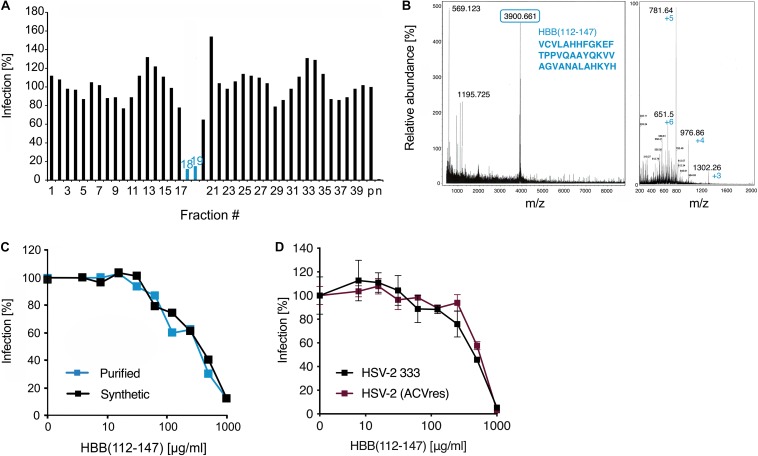
Identification of a C-terminal fragment of hemoglobin as HSV-2 inhibitor in a placenta-derived peptide library. **(A)** Fractions 1–39 of pH pool 4 of the placenta library were added to ELVIS cells and subsequently infected with HSV-2. Infection rates were determined 2 days later by quantifying β-galactosidase activities in cellular lysates. **(B)** MALDI-MS (left) and ESI-MS (right) spectra of fraction 19. The peaks correspond to HBB(112–147). **(C)** HBB(112–147) purified from placenta and chemically synthesized peptide inhibit HSV-2 infection. Peptides were added at indicated concentrations to ELVIS cells, and cells were infected with a clinical HSV-2 isolate. Infection rates were determined 2 days later by quantifying β-galactosidase activities in cellular lysates. **(D)** Activity of HBB(112–147) against a lab strain of HSV-2 (333) and a clinical, highly ACV-resistant isolate. Peptides were added to ELVIS cells at indicated concentrations and cells were infected after 30 min incubation. Infection rates were determined 24 hpi by quantifying β-galactosidase activity.

**FIGURE 2:**
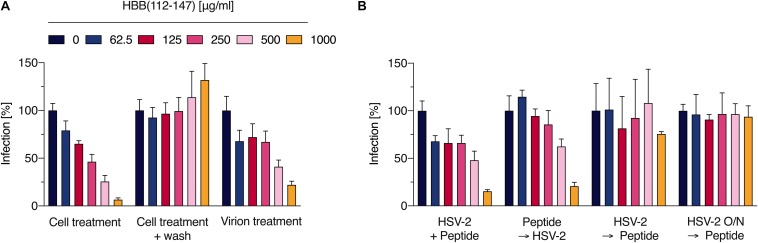
The hemoglobin fragment blocks an early step in the HSV-2 life cycle. **(A)** Indicated concentrations of HBB(112–147) were added to cells and incubated for 30 min. Thereafter, cells were either infected directly with HSV-2 (cell treatment) or washed, supplemented with fresh medium and then infected (wash). Additionally, virus was first exposed to HBB(112–147) for 3 h at indicated concentrations, and then these mixtures were used to infect cells (resulting in a 20-fold dilution of the inoculum). Infection rates were determined 2 days later by quantifying β-galactosidase activities in cellular lysates. Shown are average values derived from two experiments each performed in quadruplicates +SD. **(B)** In order to determine which stage of infection HBB(112–147) interferes with, peptide at indicated concentrations was either added to cells directly prior to infection, pre-incubated for 30 min on cells at 37°C, added 2 h post-infection (with removal of virus and addition of fresh medium), or added after overnight infection (16 hpi). Infection was evaluated by quantifying β-galactosidase activities in cellular lysates 24 hpi. Shown are average values derived from one experiment in triplicates +SD.

5,000 ELVIS cells were seeded the day before into 96-well plates. Cells were incubated with 0–1000 μg/ml of HBB(112–147) for 30 min at 37°C in triplicates prior infection with clinical isolates of HSV-1 or HSV-2. Infection rates were determined 1 or 2 day(s) later as described above. For mechanistic experiments or comparative analyses (Figures 1D, 2B) the HSV-2 strain 333 was used.

### Effect of HBB(112–147) on Zika Virus Infection

Virus stocks of ZIKV MR766, a ZIKV strain isolated from a sentinel rhesus macaque in 1947 (Dick et al., 1952) were generated by infecting Vero E6 cells. After 3–5 days the virus was collected by centrifuging the cell supernatant for 3 min at 330 g to remove cell debris. Virus stocks were stored at −80°C. Vero E6 cells were cultured in DMEM supplemented with 2.5% (v/v) heat-inactivated FCS, 2 mM L-glutamine, 1 mM sodium pyruvate, 1x non-essential amino acid, 100 units/ml penicillin and 100 μg/ml streptomycin. 6,000 Vero E6 cells were seeded into 96-well plates the day before. Cells were incubated with 0–1000 μg/ml or 0–200 μg/ml PSVBS (Schandock et al., 2017) in triplicates for 2 h at 37°C prior infection with ZIKV MR766. Two days later infection rates were determined with a cell-based ZIKV immunodetection assay. Cells were washed with PBS and fixed with 4% paraformaldehyde (PFA) for 20 min at room temperature. Cell permeabilization was performed with cold methanol for 5 min at 4°C and cells were then washed with PBS. Afterward, cells were incubated with mouse anti-flavivirus antibodies 4G2 in antibody buffer for 1 h at 37°C, washed 3 times with washing buffer and incubated with a HRP-coupled anti-mouse antibody (1:20,000) for 1 h at 37°C. After 4 washing steps with PBS TMB substrate was added. After an incubation of 5 min at room temperature, reaction was stopped with 0.5 M sulfuric acid and absorption was measured at 450 nm and baseline corrected at 650 nm using an ELISA microplate reader. Values were corrected for the background signal derived from uninfected cells and triplicates were expressed as mean ± standard error derived from two independent experiments.

### Effect of HBB(112–147) on HIV-1 Infection

Virus stocks of CCR5-tropic HIV-1 NL4-3 92TH014.12 (Papkalla et al., 2002) were generated by transient transfection of HEK293T cells as described (Münch et al., 2007a). Transfection mixture was replaced by 2 ml DMEM supplemented with 2 mM L-glutamine, 100 units/ml penicillin, and 100 μg/ml streptomycin and 2.5% heat-inactivated FCS after overnight incubation. 40 h later, virus was collected by centrifuging the cell supernatant to remove cell debris for 3 min at 330 g. Virus stocks were stored at −80°C. For the infection assays, the reporter cell line TZM-bl was used and cultured in DMEM supplemented with 2 mM L-glutamine, 100 units/ml penicillin, and 100 μg/ml streptomycin and 10% heat-inactivated FCS. 10,000 TZM-bl cells were seeded the day before into 96-well plates. The cells were incubated with 0–1000 μg/ml of HBB(112–147) or 0–50 μg/ml of heparin as control for 2 h at 37°C in triplicates prior infection with HIV-1. The TZM-bl cell line is stably transfected with an LTR-lacZ cassette and upon infection with HIV-1 the viral protein Tat is expressed, which activates the LTR and results in the β-galactosidase expression. Infection rates were determined 3 days later by detecting the β-galactosidase activity in cellular lysates using the Gal-Screen β-Galactosidase Reporter Gene Assay System for Mammalian Cells (Thermo Fisher Scientific) and the Orion II microplate luminometer (Berthold, Bad Wildbad, Germany). All values represent reporter gene activity (RLU/s) derived from triplicates minus background activities derived from uninfected cells. Triplicates are expressed as mean ± standard error derived from two independent experiments.

### Effect of HBB(112–147) on Rubella Virus Infection

40–60 plaque forming units each of the three rubella virus isolates Berlin.DE/17.97 (derived from a CRS case, genotype 1E), Bucuresti.ROU/25.03 (1G) and Rzeszow.POL/03.07 (1E) were incubated with 0–1000 μg/ml HBB(112–147) for 1 h. Mixtures were used to inoculate Vero cells. Cells were treated with CMC-Overlay and incubated at 37°C. Five days later, Rubella virus infection was quantified by an indirect immunocolorimetric assay using the monoclonal “Anti-Rubella-E1(MAB 925)” antibody as described elsewhere (Finsterbusch et al., 2009).

### Colorimetric MTT- Based Cell Viability Assay

12,000 Vero E6 cells per well were seeded in 96-well flat bottom plates in 100 μl DMEM (supplemented with 2.5% heat-inactivated fetal calf serum, 2 mM L-glutamine, 100 units/ml penicillin, 100 μg/ml streptomycin, 1 mM sodium pyruvate, and 1x non-essential amino acids) medium and incubated overnight. The next day, the medium was removed and 90 μl of X-vivo medium (supplemented with 2 mM L-glutamine and 100 units/ml penicillin, 100 μg/ml streptomycin) was added. HBB(112–147) was serially 1:2 diluted and 10 μl of each dilution was added to the cells. Cells were cultured in CO_2_ incubator at 37°C and cell viability was quantified after 48 h with the MTT-based assay. The medium was removed and 90 μl PBS and 10 μl MTT (5 mg/ml) solution were added per well. Following a 2.5 h incubation time at 37°C, supernatant was discarded and formazan crystals were dissolved in 100 μl 1:1 DMSO-EtOH solution. Absorption was measured at 450 nm and baseline was corrected at 650 nm using a Vmax kinetic microplate reader.

### Protease Digestion Experiments

Proteases used were Chymase (Sigma C8118), Cathepsin D (Sigma SRP6415), Cathepsin G (RP-77525), Cathepsin E (Biovision 7842), Pepsin (Roche 10108057001), Trypsin porcine pancreas (Sigma T0303-1G), Lysozyme from chicken egg white (Sigma L6876), Napsin A (RND 8489-NA). Digestion experiments were carried out with purified human hemoglobin (Sigma H7379) and recombinant or purified proteases. 100 μg hemoglobin (ca. 1.56 nmol) were digested with Chymase (in Tris-HCl 0.05 M, pH 8.0/0.26 M NaCl), Cathepsin D, G and E (in 0.2 M citrate buffer, pH 5.0), Pepsin (in 20 mM sodium acetate buffer, pH 3.5), Trypsin (in 0.1 M Tris–HCl, with 10 mM CaCl2, pH 8), Lysozyme (in 10 mM Tris-HCl, pH 8) or Napsin A (in 0.2 M NaCl, 0.1 M sodium acetate, pH 3.6). All proteases were used at a 1:100 molar ratio (15 pmol) and reactions were incubated at 37°C for 2 h.

### SDS-PAGE

For SDS-PAGE, samples were incubated with Protein Loading Buffer (LiCor) and reducing agent and heated. Four microgram of hemoglobin was loaded per lane on a NuPAGE^TM^ 4–12% Bis-Tris Protein Gel (Thermo Fisher). After running, the gel was washed 1 × 5 min with ultrapure water, then fixed with 50% MeOH/7% acetic acid for 15 min and washed 3 × 5 min with ultrapure water. Staining was then performed with GelCode Blue (coll oidal coomassie) overnight. Destaining was done with ultrapure water until the background appeared clear, the gel then imaged in a LiCor Odyssey system.

### Circular Dichroism (CD)

CD spectra of pre-incubated peptide samples were measured on a JASCO J-1500 spectrometer in a 1 mm High Precision Cell by Hellma Analytics. An aqueous solution (MilliQ water) of HBB(112–147) was adjusted to pH 7 or pH 5 using 1 N HCl solution with a final concentration of 0.1 mg/ml. The samples with a volume of 300 μL were measured at room temperature. The CD signal was recorded from 260 to 180 nm with a bandwidth of 1 nm. The data pitch was set to 0.2 nm, while the scanning speed was 5 nm/min. Each sample was measured in three individual scans and the data were accumulated. Spectra are baseline corrected against MilliQ water, smoothened (Means Movement, convolution width 17) and normalized. The data was processed in the software Spectra Analysis and CD Multivariate SSE by JASCO.

### Measurement of Napsin in Placenta Tissue by Dot Blot

Using a methanol activated Immobilon-FL PVDF membrane (Merck, Part# IPFL00010, LOT# R6KA6693E) detection was carried out with anti-hNapsin A (R&D Systems Affinity Purified Rabbit IgG, Part# AF8489, LOT# CJEB0115011) and beta-Actin (8H10D10) Mouse antibody (Cell Signaling Technology, LOT# 17) both 1:1000 diluted in blocking buffer over night after blocking with LiCor Odyssey Blocking Buffer (TBS) (Part# 927-50000, LOT# X2321). Detection was performed with IRDye 800 CW Goat anti-Rabbit 926-32211 (LOT# C70426-05) and IRDye 680RD Goat anti-Mouse 926-68070 (LOT# C70427-05) antibodies 1:10000 diluted for 1 h on a Li-Cor Odyssey CLx system with Image Studio (ver 5.2).

### Identification of HBB(112–147) in the HB Digested Sample by LC-ESI-MSMS

Hemoglobin digests were reduced with 5 mM DTT for 20 min at RT and subsequently alkylated with iodoacetamide for 20 min at 37°C. The samples were measured using an LTQ Orbitrap Velos Pro system (Thermo Fisher Scientific) online coupled to an U3000 RSLCnano (Thermo Fisher Scientific) uPLC as described previously (Mohr et al., 2015), with the following modifications: For separation, a binary gradient consisting of solvent A (0.1% FA) and solvent B (86% ACN, 0.1% FA) was employed. After loading onto the precolumn, the sample was concentrated and washed in 5% B for 5 min. In a first elution step, the percentage of B was raised from 5 to 15% in 5 min, followed by an increase from 15 to 40% B in 30 min. The column was washed with 95% B for 4 min and re-equilibrated for subsequent analysis with 5% B for 19 min. For visualization, spectral data was exported from the datafile using XCalibur Qual Browser 2.2 (Thermo Fisher Scientific, Bremen, Germany). Database searches were performed using PEAKs X^1^ (Zhang et al., 2012). For peptide identification, MS/MS spectra were correlated with the UniProt human reference proteome set^2^. Carbamidomethylated cysteine was considered as a fixed modification along with oxidation (M) as a variable modification. False discovery rates were set on the peptide level to 1%.

### Bacterial Strains

**Table:** 

Strain	Origin
*P. aeruginosa* BSU856	ATCC27853
*P. aeruginosa* BSU1294	Clinical isolate, Ulm collection
*P. aeruginosa* BSU1295	Clinical isolate, Ulm collection
*P. aeruginosa* BSU1455	Clinical isolate, Ulm collection
*P. aeruginosa* BSU1456	Clinical isolate, Ulm collection
*P. aeruginosa* BSU1457	Clinical isolate, Ulm collection
*P. aeruginosa* BSU1458	Clinical isolate, Ulm collection Carbapenem resistant (Hagemann et al., 2018)

### Radial Diffusion Assay

Bacteria were cultured at 37°C in a 5% CO_2_ atmosphere overnight, pelleted by centrifugation and washed in 10 mM sodium phosphate buffer. Following resuspension in 10 mM sodium phosphate buffer optical density was determined at 600 nm (OD_600 *nm*_). 2 × 10^7^ bacteria were seeded into a petri dish in 1% agarose dissolved in 10 mM sodium phosphate buffer. After cooling at 4°C for 30 min 2–3 mm holes were punched into the 1% agarose. Peptides adjusted to the desired concentration in 10 μl of ddH_2_O were filled into the agar-holes. Following incubation at 37°C in ambient air for 3 h plates were overlaid with 1% agarose solution containing 3% tryptic soy broth (TSB) dissolved in 10 mM phosphate buffer. Inhibition zones in cm were determined following 16–18 h incubation time at 37°C in a 5% CO_2_ atmosphere and used to evaluate antibacterial potency. For LPS inhibition experiments, 62.5 μg/ml HBB(112–147) was incubated for 1 h at 37°C together with varying concentrations of LPS of *P. aeruginosa* 10 (Sigma L9143) followed by the same procedure as described above.

### Survival Assay

*P. aeruginosa* BSU856 cells were grown in THY broth [Todd-Hewitt Broth (Oxoid) supplemented with 0.5% yeast extract (BD, Miami, United States)] at 37°C in a 5% CO_2_ atmosphere overnight. 1 ml of the culture adjusted to an OD_600__*nm*_ of 0.1 was centrifuged and the bacterial pellet was resuspended in 1 ml of assay medium (5% TSB dissolved in 0.9% NaCl adjusted to different pH values). 90 μl microliter of the bacterial suspension was mixed with 10 μl of HBB(112–147) of the desired concentration or with 10 μl of ddH_2_O (negative control) followed by incubation at 37°C. Samples were taken at the indicated time points, dilutions were prepared and platted on sheep blood agar plates (TSA + SB, Oxoid, Basingstoke, United Kingdom). Colony Forming Units (CFU) were counted after overnight incubation of the agar plates at 37°C in a 5% atmosphere and the survival of the cells was calculated in comparison to the CFU present at the beginning of the experiment (*t* = 0 min) and normalized to the mock treated bacterial sample grown in the corresponding assay medium.

### SYTOX Green Membrane Permeabilization Assay

An equivalent of 100 μl of an OD_600 *nm*_ of 0.1 of mid-exponential *P. aeruginosa* BSU856 cells were harvested by centrifugation and resuspended in 5% TSB dissolved in 0.9% NaCl adjusted to three different pH values (pH 7, pH 5.5, pH 4.5). The samples were treated with HBB(112–147) of different concentrations or were mock treated with ddH_2_O. After incubation at 37°C for 1 h, the cells were pelleted and resuspended in 5% TSB dissolved in 0.9% NaCl adjusted to pH 7 containing 0.2 μM SYTOX green stain. The fluorescent intensity of the samples was measured in a Tecan infinite M200 plate reader with an excitation wavelength of 488 nm and an emission wavelength of 530 nm. The relative fluorescence of the samples was calculated by normalizing the peptide treated samples to the mock treated samples grown in the corresponding assay medium.

### Transmission Electron Microscopy

*P. aeruginosa* BSU856 cells, grown till mid-exponential growth phase, were harvested by centrifugation and resuspended in 5% TSB dissolved in 0.9% NaCl (pH 4.5). 5 × 10 (Mor and Kwon, 2015) cells were treated with 0.1 mM HBB(112–147) for 1 h at 37°C followed by centrifugation. The pelleted cells were fixed with 2.5% glutaraldehyde containing 1% saccharose in phosphate buffer (pH 7.3). Samples were washed five times with phosphate buffer and post-fixed in 2% aqueous osmium tetroxide. After dehydrating the samples in a graded series of 1-propanol, they were blockstained in 1% uranyl acetate and embedded in Epon. Ultra-thin sections (80 nm) were collected on copper grids, contrasted with 0.3% lead citrate for 1 min and imaged in a Zeiss TEM 109 or in a Jeol TEM 1400.

### Evolutionary Conversation of HBB(112–147) Sequence in Hemoglobin

The following orthologs of the hemoglobin beta globin chain were included in the sequence analysis (numbers give accession numbers): Homo sapiens (Human) P68871; Rattus norvegicus (Rat) P02091; Mus musculus (Mouse) P02088; Sus scrofa (Pig) P02067; Oryctolagus cuniculus (Rabbit) P02057; Pan troglodytes (Chimpanzee) P68873; Bos taurus (Bovine) P02070; Mesocricetus auratus (Golden hamster) P02094; Ovis aries (Sheep) P02075; Macaca fascicularis (Cynomolgus monkey) P68223; Panthera pardus orientalis (Amur leopard) P04244; Capra hircus (Goat) P02077; Bradypus tridactylus (Pale-throated three-toed sloth) P14526; Ailuropoda melanoleuca (Giant panda) P18983; Dasypus novemcinctus (Nine-banded armadillo) P02087; Macropus eugenii (Tammar wallaby) Q6H1U7; Tachyglossus aculeatus aculeatus (Australian echidna) P02110; Xenopus laevis (African clawed frog) P02132; Chelonoidis carbonarius (Red-footed tortoise) Q98905; Meleagris gallopavo (Common turkey) P81023; Gallus gallus (Chicken) P02112; Danio rerio (Zebrafish) Q90486;

Protein sequences were aligned using Clustal W^3^ and residue conservation was determined using the Scorecons Server^4^. Shannon’s information theoretical entropy was applied to measure the diversity of amino acids at a specific site. Residues were classified into one of seven types: aliphatic (AVLIMC), aromatic (FWYH), polar (STNQ), positive (KR), negative (DE), special conformations (GP) and gaps. This convention follows that of Mirny and Shakhnovich (1999). Statistical calculations were performed with a two-tailed unpaired Student’s *t*-test using Graph Pad Prism Version 5.0.

## Results

### Identification of Placenta-Derived Peptide Fractions That Simultaneously Reduce HSV-2 Infection and Growth of *P. aeruginosa*

In an attempt to identify endogenous antiviral and antibacterial peptides in placenta, we generated a peptide library from 10 kg of pooled homogenized placental tissue. Human tissue was obtained from healthy individuals in a maternity ward of a local hospital and was processed immediately after delivery. Peptides were extracted by an ice-cold acetic acid extraction procedure and the obtained peptide and protein fractions were separated by ultrafiltration (cut-off: 50 kDa). The peptide fraction was then chromatographically separated and resulted in approx. 250 different peptide containing fractions that were used for bioscreening (Liepke et al., 2003). To identify peptides with anti-HSV-2 activity, all fractions of pH pools 0–4 were dissolved in PBS and added to ELVIS^TM^ cells, an HSV-2 reporter cell line expressing β-galactosidase upon viral infection. Thereafter, cells were infected with HSV-2 and infection rates determined 2 days later (before occurrence of viral CPE) by quantifying β-galactosidase activity in cellular lysates. Only fraction 8 of pH pool 2 and fractions 18–20 of pH pool 4 markedly suppressed HSV-2 infection (Supplementary Figure S1A). To confirm these results, a twice as high concentration of these fractions was tested again for anti-HSV-2 activity. Antiviral activity of fraction 8 of pH pool 2 could not be confirmed (not shown), however, fractions 18 and 19 inhibited HSV-2 infection by ∼80% (Figure 1A). Interestingly, the same fractions of pH pool 4 were also the most potent inhibitors of growth of *P. aeruginosa* (Supplementary Figure S1B).

Subsequent mass spectrometric analysis by MALDI-MS revealed a predominant peptide with a mass of 3902.5 Da (Figure 1B). Edman sequencing allowed to determine the N-terminal sequence starting with VCVLA… and database search identified the corresponding peptide: a 36-mer derived from the C-terminal region of the hemoglobin subunit β (HBB) encompassing residues 112–147 (Figure 1B, numbering according to UniProt sequence P68871). The same peptide was previously isolated by us from a similar placenta library and shown to exert antibacterial activity against Gram-positive and -negative bacterial species (Liepke et al., 2003). However, no antiviral activity was reported. Thus, we next determined the anti-HSV-2 activity of placenta-purified HBB(112–147) and that of the chemically synthesized version. As shown in Figure 1C, both peptides resulted in a dose-dependent inhibition of HSV-2 infection with IC_50_ values in the median μg/ml range, without causing cytotoxic effects (Supplementary Figure S2). Thus, placenta contains a C-terminal hemoglobin β fragment that restricts growths of bacteria and inhibits HSV-2 infection. As resistance to conventional anti-HSV drugs such acyclovir (ACV) is a growing problem especially with HSV-2 in immunocompromised patients (Piret and Boivin, 2011), we investigated whether HBB(112–147) is also effective against ACV-resistant HSV-2. Indeed, a highly ACV-resistant clinical HSV-2 isolate was equally inhibited by the peptide as the HSV-2 333 lab strain (Figure 1D).

### HBB(112–147) Inhibits HSV-2 Infection by Blocking Viral Entry

We next analyzed the antiviral mechanism of HBB(112–147). For this, cells were exposed to increasing concentrations of the peptide for 30 min. Thereafter, cells were either infected directly with HSV-2 (cell treatment), or washed and subsequently infected (wash). Additionally, virions were first treated with the peptide for 3 h at indicated concentrations, and then used to infect cells, resulting in 20-fold reduced concentrations of HBB(112–147) in cell culture (virion treatment). No inhibitory activity was observed if peptide was removed by washing prior to infection, suggesting that the hemoglobin fragment needs to be present during infection and that it does not induce an antiviral state of cell (Figure 2). If added 30 min prior to infection, HBB(112–147) dose-dependently reduced infection with an IC_50_ of ∼200 μg/ml, confirming results shown above (Figure 2) and demonstrating that it blocks an early step in the viral life cycle. Interestingly, exposure of the virions prior to infection also suppressed infection (virion treatment). The two highest concentrations tested, 500 and 1000 μg/ml, reduced HSV-2 infection by 60 and 78%, respectively. These concentrations correspond to final cell culture concentrations of 25 and 50 μg/ml, that hardly reduce infection, suggesting that HBB(112–147) seems to interact with the HSV-2 particles in a way that prevents infection. Consistent with an entry-inhibiting effect, HBB(112–147) only exerted potent inhibition if added to cells prior to infection (directly or with 30 min pre-incubation), but did not inhibit HSV-2 infection if added 2 or 16 h after infection (Figure 2B). This is consistent with inhibition by Heparin, a well-described HSV entry inhibitor preventing virion adsorption (Supplementary Figure S3).

### HBB(112–147) Does Not Affect HSV-1, HIV-1, Zika and Rubella Virus Infection

**FIGURE 3:**
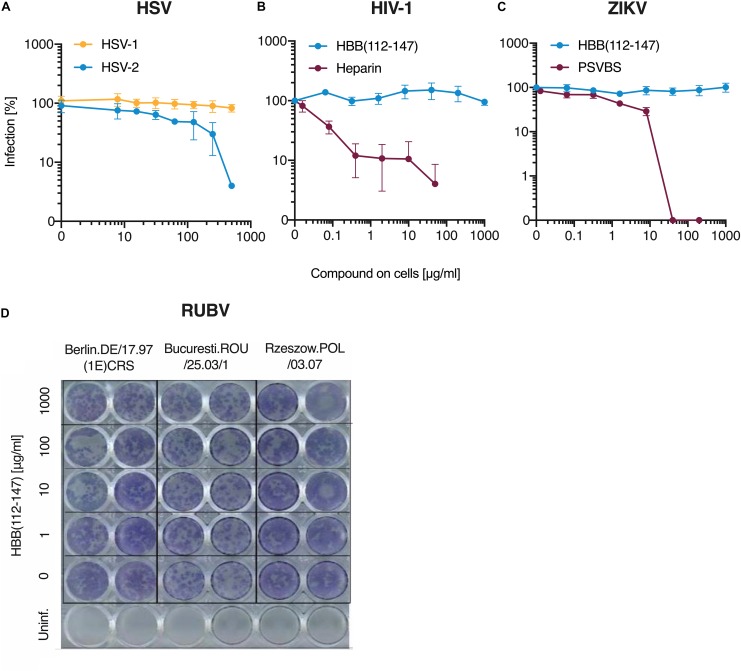
Antiviral activity of HBB(112–147) against HSV-1, HIV-1, ZIKV and RUBV. **(A)** Peptide was added to ELVIS cells and cells then infected with one clinical HSV-1 isolates and a second isolate of HSV-2. Infection rates were determined two days later by quantifying β-galactosidase activities. **(B)** TZM-bl reporter cells were incubated with HBB(112–147) or the polyanion Heparin and then infected with HIV-1. Three days post-infection, infection was determined by measuring β-galactosidase activities in cellular lysates. **(C)** Vero E6 cells were supplement with HBB(112–147) or the macromolecular compound PSVBS and infected with ZIKV-MR766. Two days later, infection was quantified by an in cell ELISA that detected the viral E protein. Values shown in **(A–C)** are mean values derived from triplicate experiments. **(D)** Three rubella virus isolates were exposed to indicated concentrations of HBB(112–147) and then inoculated onto Vero cells. Cells were treated with CMC-Overlay and incubated at 37°C. Five days later, RUBV infection was quantified by an indirect immunocolorimetric assay using the monoclonal anti-Rubella-E1(MAB 925) and photos taken by a digital camera.

We next tested whether the hemoglobin fragment may also inhibit HSV-1 infection. Surprisingly, HBB(112–147) did not block infection of a clinical HSV-1 isolate whereas it was active against a second HSV-2 isolate tested (Figure 3A). We next determined the effect of the peptide on viruses for which dia-placental transmissions have been reported. HBB(112–147) and the polyanion heparin as control (Shieh et al., 1992) were titrated on TZM-bl cells, an HIV-1 reporter cell line that expresses β-galactosidase upon infection. As shown in Figure 3B, the polyanion inhibited HIV-1 infection in a dose-dependent manner whereas HBB(112–147) was inactive. Likewise, no inhibition of Zika Virus (ZIKV) infection of Vero cells was observed, as shown by immunodetection assay using a flavivirus antibody-based ELISA (Müller et al., 2018), whereas the macromolecular polyanion PSVBS (Schandock et al., 2017) was active (Figure 3C). For both ZIKV and HIV-1, even virus treatment with up to 1 mg/ml HBB(112–147) on virus did not result in any inhibition, while the molecular tweezer CLR01 resulted in destruction of both virions as previously published (Supplementary Figure S4). Similarly, no antiviral effect of HBB(112–147) against three different Rubella virus (RUBV) isolates, one isolated from a patient with congenital rubella syndrome, were observed, as shown by indirect immunocolorimetric detection of RUBV infection in Vero cells (Figure 3D). Thus, HBB(112–147) is no broad-spectrum antiviral agent but rather a specific inhibitor of HSV-2.

### Proteolytic Generation of HBB(112–147)

**FIGURE 4:**
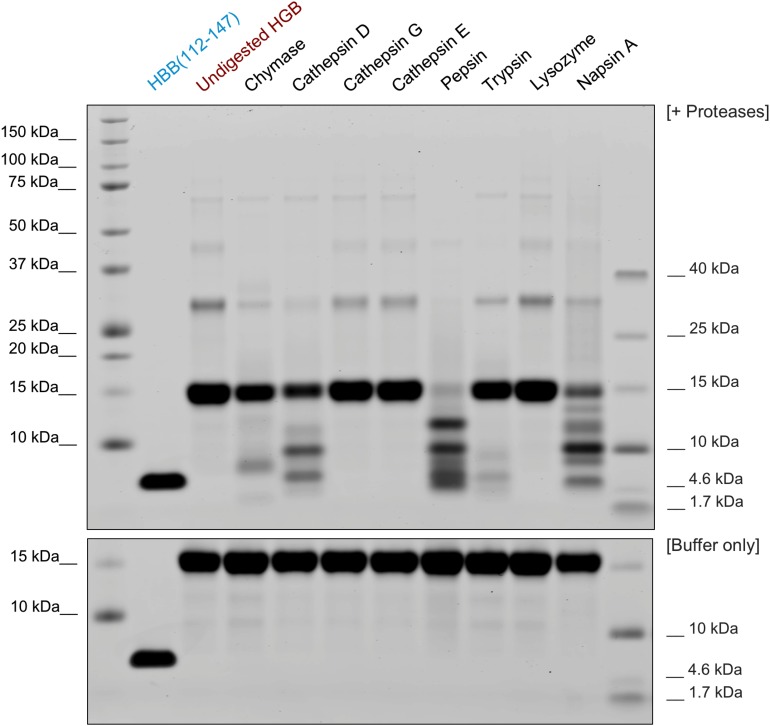
Generation of HBB(112–147) by proteolytic digestion. Purified human hemoglobin was incubated with several proteases (at 1:100 molar ratio) and digested for 2 h at 37°C or incubated with the respective buffer only for the same time (bottom). The reactions were then separated by SDS-PAGE and total protein stained by colloidal coomassie. For the bottom panel, human hemoglobin was incubated in the respective digestion buffers without addition of protease and separated as well.

In adults, the most common hemoglobin type is a tetramer called hemoglobin A, consisting of two α and two β subunits that are non-covalently bound (α2β2). In this tetrameric state, the putative protease cleavage site in the β subunits at positions L111 and V112 is not solvent exposed and hence not accessible for proteases (Supplementary Figure S5). However, α2β2 rapidly dissociates into respective dimers and monomers at acidic pH (Huang et al., 2013). Thus, we analyzed whether HBB(112–147) can be generated under low pH conditions by acidic proteases. In order to determine which proteases might be responsible for the proteolytic liberation of HBB(112–147) from full-length hemoglobin, purified human hemoglobin was subjected to digestion by a panel of 8 proteases. Digestion with Chymase, Cathepsin D as well as Pepsin, Trypsin and Napsin A (Figure 4) resulted in lower signal for uncleaved monomeric globin (16 kDa) and generation of smaller fragments. As bands consistent with a size of HBB(112–147) at approximately 4 kDa were especially observed for digestions with Cathepsin D, Pepsin and Napsin A, those digestions were also subjected to mass spectrometric analysis in order to confirm generation of HBB(112–147). Indeed, Cathepsin D and Napsin A-digested hemoglobin yielded a signal consistent with generation of HBB(112–147) (Supplementary Figure S6). No such signal was detected in Pepsin-digested hemoglobin (not shown), suggesting generation of another peptide fragment with similar size. In kinetic digestion experiments with Cathepsin D, a fragment running at the same height as synthetic HBB(122–147, 3902.5 kD) was observed already at the first time point (20 min), while no fragments were generated in a buffer-only mock digestion (Supplementary Figure S7). As Napsin A has not been previously described to be expressed in placental tissue, we analyzed a placenta homogenate by dot blot analyses using a Napsin A specific antibody. As shown in Supplementary Figure S6, Napsin A is readily detectable in the placenta homogenate (Supplementary Figure S8).

### Conservation Score and Biophysical Characterization of HBB(112–147)

**FIGURE 5:**
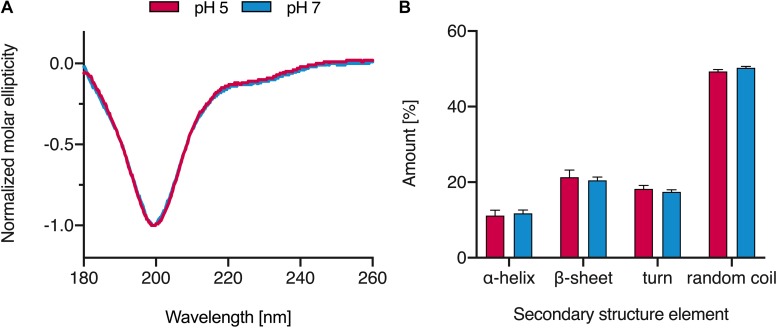
CD spectra of HBB(112–147). **(A)** The peptide was dissolved in MilliQ water at pH 5 and 7 and CD spectra were recorded. **(B)** Secondary structure elements of HBB(112–147) based on the analysis of CD spectra results; spectra were baseline corrected against MilliQ water, smoothened and normalized. Values shown were derived from triplicate measurements (+SD).

To determine the evolutionary conservation of the HBB(112–147) peptide sequence, we aligned the β-hemoglobin orthologs of 22 vertebrate species (Supplementary Figure S9, bottom right) and determined the conservation score, i.e., the diversity of amino acids at each site (Supplementary Figure S9, top). The average conservation score of residues within HBB(112–147) was not significantly different from that outside this region (Supplementary Figure S9, bottom left). This suggests that the HBB(112–147) sequence is evolutionarily not more conserved than the remainder of the β-hemoglobin protein. Analysis of the HBB(112–147) sequence by ProtParam tool revealed a positive isoelectric point (pI) of 9.05, a net excess of two positively charged residues, and an estimated half-life of >100 h in mammalian reticulocytes, characterizing the peptide as very stable. We determined the CD spectra of HBB(112–147) at neutral and acidic pH. As shown in Figure 5, the peptide adopts mainly a random coil structure with modest contribution of other structures while the content of secondary structures is not changed significantly if the peptide is dissolved in solutions with acidic or neutral pH. This lack of ordered structures and high random coil content could be an indication of intrinsically disordered structures (Chouard, 2011; Wright and Dyson, 2015). Several antimicrobial peptides (AMPs) are known to form disordered regions that could adopt more ordered states upon binding to their cellular targets (Dyson and Wright, 2005; Sugase et al., 2007; Chouard, 2011; Wright and Dyson, 2015). However, the functional role of these intrinsically disordered proteins has been recognized only recently (Dyson and Wright, 2005), and further analysis and structure mining would be necessary to clarify whether the high random coil content of HBB(112–147) could change upon interaction with its target.

### HBB(112–147) Inhibits Growth of Several Clinical Isolates of *Pseudomonas aeruginosa*

**FIGURE 6:**
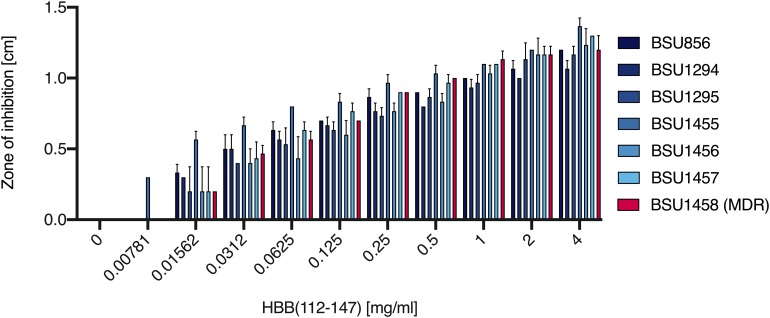
Antibacterial activity of HBB(112–147) against clinical *Pseudomonas aeruginosa* isolates. The effect of indicated concentrations of HBB(112–147) was tested against several clinical isolates of *Pseudomonas aeruginosa* in a radial diffusion assay. BSU1458 (red) is a carbapenem/multi-drug resistant *P. aeruginosa* isolate. Depicted are inhibition zones in cm observed in a radial diffusion assay (*n* = 3, mean values +SD).

*P. aeruginosa* is a frequent nosocomial pathogen causing many life-threatening infections that are often complicated through the worldwide occurrence of multi-resistant strains. To assess if placental tissue may represent a source for AMPs exhibiting anti-pseudomonal activity we screened a peptide library against *P. aeruginosa* strain ATCC27853. One of the fractions with the highest activity against *P. aeruginosa* (fraction 19) contained large amounts of HBB(112–147) (Supplementary Figure S1B). To substantiate these results and to confirm that the antimicrobial compound in this fraction was HBB(112–147), various clinical *P. aeruginosa* isolates and strain ATCC27853 were evaluated for susceptibility against this purified hemoglobin fragment in radial diffusion assays. All of the strains investigated demonstrated a susceptibility to HBB(112–147) in a dose dependent manner down to concentrations as low as than 15.6 μg/ml (Figure 6). To explore if HBB(112–147) is also effective against a multiresistant strain of this species, we determined HBB(112–147) susceptibility of a carbapenem resistant *P. aeruginosa* strain, that had previously shown antibiotic susceptibility only to Colistin, a toxic, last resort antibacterial compound (Hagemann et al., 2018). Inhibition zones for this strain were indistinguishable from values obtained for other clinical *P. aeruginosa* isolates (Figure 6, red column).

### The Antibacterial Effect of HBB(112–147) Is pH Dependent

**FIGURE 7:**
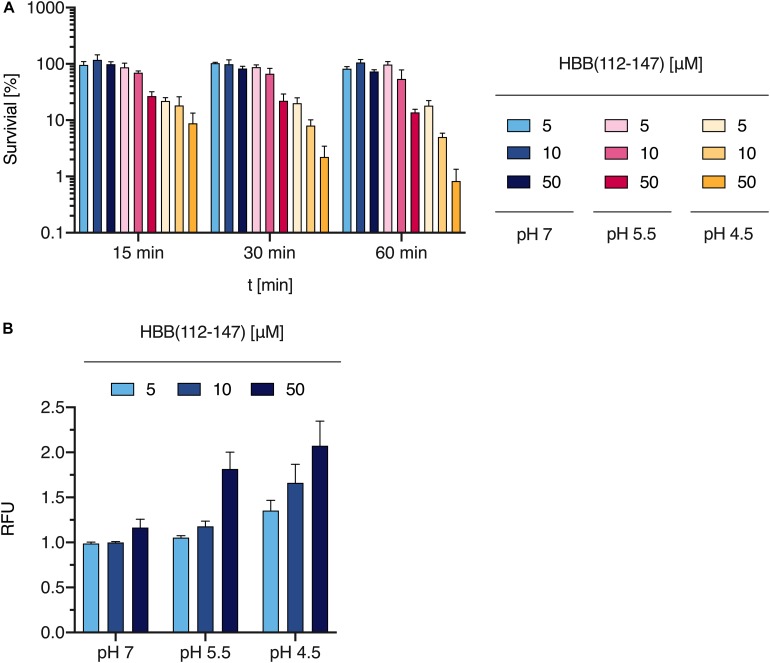
Effect of HBB(112–147) on bacterial survival and membrane integrity. **(A)** The effect of HBB(112–147) on *P. aeruginosa* growth was quantified in a survival assay. 5, 10, and 50 μM of the peptide were added to an overnight culture of *P. aeruginosa* at pH values of 7, 5.5, and 4.5 for 15, 30, and 60 min. Bacteria were quantified by CFU determination. Values are expressed as % of growth controls not exposed to HBB(112–147). Data shown are average values derived from three biological replicates +SD. **(B)** Plasma membrane damage of *P. aeruginosa* following HBB(112–147) treatment for 1 h at 37°C, was tested at indicated pH values by uptake of SYTOX green and subsequent quantification of fluorescence in a Tecan infinite M200 plate reader. Shown are mean values and SD of *n* = 3. Values shown are relative fluorescence units (RFU).

Many antimicrobial peptides show highest antibacterial activity at a pH below 7 (Malik et al., 2016) and isolation of HBB(112–147) from placental tissue was carried out under acidic conditions. Furthermore slightly acidic conditions are present in the radial diffusion assay that we used for screening of the placental library as well as for the evaluation of the susceptibility of *P. aeruginosa* against HBB(112–147). To investigate a potential pH dependency of the antimicrobial effect of HBB(112–147), bacterial survival assays were carried out under different pH conditions. An overnight culture of *P. aeruginosa* was resuspended to an OD of 0.1 and adjusted to pH values of 4.5, 5.5, and 7. Following exposure to 5–50 μM concentrations of HBB(112–147) bacterial survival was quantified through CFU measurements. After 15 min at pH 4.5 less than 10% of the *P. aeruginosa* cells growing in control tubes of the same pH without HBB(112–147) were still viable, a value which dropped to less than 1% after 60 min (Figure 7A). At pH 7 little if any growth inhibition could be observed when compared to the appropriate controls even after 60 min at 50 μM concentration of HBB(112–147) (Figure 7A).

### HBB(112–147) Destroys the Membrane Integrity of *P. aeruginosa*

**FIGURE 8:**
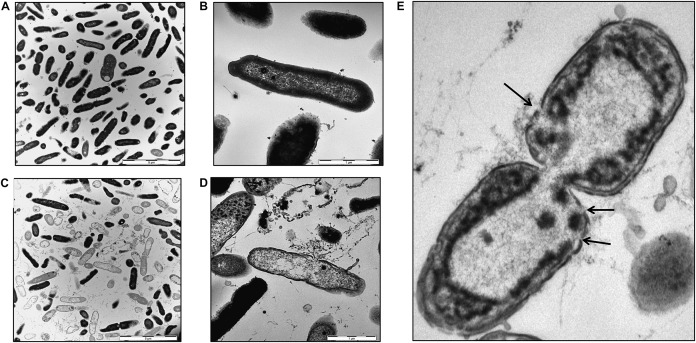
Transmission electron microscopy images of *P. aeruginosa*. Bacterial cells were grown to midlogarithmic phase and exposed to buffer **(A,B)** or 0.1 mM HBB(112–147) at pH 4.5 for 1 h **(C,D)**. Cells were fixed with 2.5% glutaraldehyde and dehydrated in a graded series of 1-propanol. Following embedding in Epon, ultra-thin sections (80 nm) were collected on copper grids, and imaged in a Zeiss TEM 109 or in a Jeol TEM 1400. Areas showing loss of membrane integrity are indicted by black arrows **(E)**. Scale bars are 5 μm **(A,C)** and 1 μm **(B,D)**.

Bacterial membrane damage is one of the most common modes of action for AMPs. To elucidate if this is also the relevant antimicrobial mechanisms for HBB(112–147) against *P. aeruginosa*, we investigated bacterial membrane integrity via SYTOX green uptake into bacterial cells. Intracellular SYTOX green enrichment and staining of nucleic acids occurs only upon disruption of bacterial membranes. Following exposure of HBB(112–147) for 1 h at 37°C, membrane integrity was compromised already at 5 μM concentrations for pH 4.5 and showed a clear dose dependent effect (Figure 7B). In support of the results obtained for the antibacterial effect, also membrane permeabilization was pH dependent. At pH 7 even at a 50 μM concentration of HBB(112–147) hardly any SYTOX enrichment could be detected in *P. aeruginosa*. To visualize loss of membrane integrity transmission electron microscopy (TEM) was performed. Treatment of *P. aeruginosa* cells was carried out with 0.1 mM HBB(112–147) for 1 h at pH 4.5. Subsequent TEM pictures revealed the presence of multiple lysed bacterial cells and leakage of intracellular content (Figures 8C,D), while controls appeared intact (Figures 8A,B). Areas of disrupted bacterial membranes are clearly visible in TEM (Figure 8E) of bacterial cells exposed to HBB(112–147). To further investigate bacterial surface molecules of *P. aeruginosa*, that might interact directly with HBB(112–147), we performed a preincubation of HBB(112–147) with *P. aeruginosa* LPS for 1 h. Subsequent testing of antimicrobial activity in radial diffusion assays, showed a dose dependent reduction of the inhibitory effect (Supplementary Figure S10), indicating that HBB(112–147) binds to LPS.

## Discussion

In the present study, we screened a placental peptide library for antiviral agents and identified a C-terminal fragment of β-hemoglobin as specific inhibitor of HSV-2 infection. This peptide, termed HBB(112–147) has previously been isolated from the same source as broad-spectrum antibacterial agent (Liepke et al., 2003). Here, we confirm and expand these findings and demonstrate that HBB(112–147) is also active against clinical isolates of *P. aeruginosa*, including a multiple-drug resistant strain (Hagemann et al., 2018). Our novel data also show that the peptide ruptures the bacteria cell wall in a pH-dependent manner resulting in release of the cytosol and consequently cell death. Moreover, we show that HBB(112–147) is generated from tetrameric hemoglobin by ubiquitously expressed aspartic proteases under acidic conditions, which are a hallmark of inflammatory tissues.

HBB(112–147) is the predominant peptide in those fractions of the peptide library (fr. 18 and 19 of pH pool 4) that inhibited HSV-2 and *P. aeruginosa*. Peptide purified from placenta and the synthetic version showed comparable antiviral activity, strongly suggesting that only the HBB(112–147) fragment but no other peptide or protein are responsible for the observed antiviral activity in these fractions. Unlike its broad antibacterial activity against various Gram-positive and -negative bacteria (Liepke et al., 2003), HBB(112–147) selectively inhibits HSV-2 but no other viral pathogen, suggesting a different antibacterial and antiviral mechanism. In fact, we show that HBB(112–147) has direct bacterial cell killing activity by inducing ruptures in the bacterial cell wall. We also demonstrate that HBB(112–147) interacts with HSV-2 particles in a way that prevents viral entry, whereas no inhibition of other enveloped viruses (HIV-1, ZIKV, RUBV, and HSV-1) was observed. Virion pre-treatment of HSV-2 did not yield overall higher inhibition rates, indicating that the effect is not due to virion disruption. As IC_50_ were nonetheless substantially lower in virion- compared to cell-treatment mode, we propose HBB(112–147) to interfere with the interaction of viral glycoproteins with a cellular receptor. It cannot be excluded that non-protein structures of the viral envelope (lipids, carbohydrates) might also be targets of HBB(112–147). However, infection-inhibitory membrane interactions would likely result in membrane disruption (as e.g., for CLR01 used in Supplementary Figure S4) and are not consistent with the overall higher inhibition observed upon cell treatment (Figure 2).

HSV-1 and HSV-2 are closely related viruses with a high sequence homology of 83% in the protein-coding regions (Dolan et al., 1998), which mediate cellular attachment and entry. The differential activity of HBB(112–147) against HSV-1 and HSV-2 may reflect differences in the glycoprotein sequence and/or composition of both viruses. In fact, both viruses differ in their sensitivity to polyanions (Hutton et al., 1973) and polycations (Langeland et al., 1987, 1988), differential binding to heparan sulfate (HS)/heparin chains (Herold et al., 1996; Trybala et al., 2000) and stimulation of viral infection in glycosaminoglycan-deficient cells by dextran sulfate (Dyer et al., 1997). Collectively these data suggest that HBB(112–147) may interact with an HSV-2 specific glycoprotein that is important for viral attachment and/or entry. The fact that antibacterial and antiviral action is exerted via a different mechanism is likely due to conformational changes of HBB(112–147) upon encounter of LPS on the surface of *P. aeruginosa* as it has been described for several other AMPs (Pulido et al., 2012). The subsequent rearrangements and membrane disruption may thus only occur at bacterial membranes and not the surface of virions. Whether interaction with viral surfaces leads to other conformation changes or the peptide is active as a random coil in this scenario remains to be studied.

As a common sexually transmitted infection, HSV-2 has a relatively high seroprevalence of 10–30% in the US and Europe and up to 30–80% in the female population of some developing countries (Malkin, 2004; Weiss, 2004; Looker et al., 2015). As oral HSV-1 infection is typically already acquired during childhood or adolescence, it is much more prevalent at ca. 60–90% seropositivity in developed countries (Smith and Robinson, 2002). While infection with either virus is often either asymptomatic or results in characteristic skin lesions, severe outcomes such as herpes simplex encephalitis (HSE) in neonates or aseptic meningitis in adults are also observed (Berger and Houff, 2008). Neonatal HSE is a particularly severe disease caused by HSV-2 infection in 70% of cases, leading to CNS involvement in 70% of cases and death in 60% of cases if untreated (CPSP, 2002). Interestingly, vertical transmission of HSV-2 from mother to child happens perinatally in the majority of cases, while only 5% of transmission occur *in utero* (Kimberlin, 2007). Despite relatively high shedding rates (compared to HSV-1) in infected women (Scoular, 2002), vertical HSV transmission is generally a rare event, occurring in up to 1/3,000 live births (Brown et al., 2005) and HSV-2 is responsible for the majority of those infections (Avgil and Ornoy, 2006). Multiple factors affect these low transmission rates: the lack of HSV receptor expression on syncytiotrophoblasts (Koi et al., 2002), transplacental transmission of protective antibodies (Brown et al., 2005) as well as low viremia in most HSV-2 patients in absence of primary infection. Despite these factors, one clinical study found HSV-2 DNA in 9% of maternal-side placenta tissue with 39% of these cases also testing positive for HSV-2 DNA on the fetal side; notably despite lack of symptoms in any of the patients at delivery (Finger-Jardim et al., 2014). Congenital infection with other herpesviruses is substantially more common: CMV infection occurs in 0.2–2.2/100 live births, while congenital VZV infection is reported with an incidence of up to 0.4–9/100 births. Notably, these numbers account for congenital infections, meaning *in utero* transmission of the virus, which is a minor occurrence for HSV-2. Considering the potent inhibition of HSV-2 infection by HBB(112–147) and the abundance of the precursor in placental tissue, a role of the peptide in preventing vertical transmission of HSV-2 *in utero* seems plausible and further experiments are needed to confirm its role *in vivo*.

*P. aeruginosa* is listed among the six bacterial species summarized as ESKAPE pathogens that pose an increased health risk to patients due to their common multidrug resistance profiles (Pendleton et al., 2013). It is responsible for a wide variety of nosocomial infections and the major bacterial agent in chronic lung infections of cystic fibrosis patients (Malhotra et al., 2019). In cystic fibrosis as well as in other serious *P. aeruginosa* caused infections the steady increase of multi-resistant *P. aeruginosa* strains severely limits therapeutic options. Often highly toxic substances such as Colistin represent the only therapeutic options for multidrug resistant strains (Hagemann et al., 2018). To address this alarming clinical problem, we used the same peptide library originating from human placenta to identify antimicrobial substances targeting *P. aeruginosa*. Interestingly the fractions exhibiting the highest antimicrobial activity against *P. aeruginosa* were also the most active fractions against HSV-2. While screening was carried out with an ATCC laboratory strain, subsequent determination of antimicrobial activity was done on *P. aeruginosa* clinical isolates including a multidrug resistant carbapenemase expressing strain (Hagemann et al., 2018). Susceptibility to HBB(112–147) showed a dose dependent effect and proved to be very similar in all of the clinical isolates we tested. Interestingly, many AMPs display antimicrobial activity preferably at low pH conditions. The survival rates of *P. aeruginosa* in the presence of HBB(112–147) were greatly affected by pH and showed the highest activity of HBB(112–147) at a pH of 4.5. Acidic pH values are found in various host niches such as the vaginal tract, the skin, the stomach, intracellularly in lysosomal compartments and at sites of bacterial infections. Many of the well-known human AMPs such as lactoferrin, LL37, dermicidin, kappacins and histatins show pH dependent antimicrobial activities (Malik et al., 2016).

Indeed, biophysical analysis of HBB(112–147) revealed features characteristic for many AMPs. This class of innate host defense molecules are 12–50 amino acids, include two or more positively charged residues provided by arginine, lysine or, in acidic environments, histidine, and a large proportion (generally > 50%) of hydrophobic residues (Sitaram and Nagaraj, 2002; Papagianni, 2003; Dürr et al., 2006). HBB(112–147) is a 36-mer with three positively charged residues (K10, K22, K34), and additionally four histidine residues, which may explain the increased antibacterial activity of HBB(112–147) under acidic conditions. Conformational changes of AMPs leading to an increase of alpha helical structures occur at low pH and represent a hallmark of pore forming AMPs. Under low pH conditions protonation of histidine residues occurs in some AMPs (Kacprzyk et al., 2007) and promotes conformational changes that improve their capacity for insertion into bacterial membranes and pore formation (Khatami et al., 2014). Many AMPs are unstructured in free solution [as HBB(112–147) is], and fold into their final configuration upon partitioning into biological membranes resulting in the disruption of bacterial membrane integrity. Especially for pH dependent AMPs, bacterial pore formation is the preferred molecular mechanism (Malik et al., 2016). Based on our SYTOX experiments that detected pH-dependent membrane damage, bacterial lysis seems to occur in *P. aeruginosa* upon exposure to HBB(112–147). This interpretation is supported by the analysis of TEM pictures that shows defects of bacterial membranes as well as spilling of bacterial intracellular content and a large proportion of apparently lysed bacterial cells. To achieve bacterial lysis, HBB(112–147) needs to interact with bacterial surface structures. LPS has previously been implicated to interact with HBB(112–147) (Liepke et al., 2003), however, if this interaction could be important in regard to the antimicrobial properties of HBB(112–147) has not been investigated. We could show here that the preincubation of HBB(112–147) with soluble *P. aeruginosa* LPS reduced its antibacterial activity in a dose dependent manner, most likely through competitive inhibition. The cationic and hydrophobic nature of many AMPs facilitates the interaction with negatively charged bacterial molecules such as LPS. Several antimicrobial peptides are known to interact with LPS, targeting AMPs to the bacterial cell surface as well as exerting an anti-inflammatory effects by blocking host reactions like cytokine release (Pulido et al., 2012). Based on the data we obtained, HBB(112–147) appears to have a similar mode of action and can be classified as typically cationic AMP.

The prerequisites for the generation of HBB(112–147) – the hemoglobin precursor and the peptide releasing protease Cathepsin D – are given almost everywhere in the body. We show that HBB(112–147) is released under acidic conditions by Cathepsin D, an aspartic endo-protease that is ubiquitously distributed in lysosomes of all cells (Barrett and Cathepsin, 1970). The main function of Cathepsin D is to degrade proteins and activate precursors of bioactive proteins in pre-lysosomal compartments (Diment et al., 1989). However, Cathepsin D can also be found in the extracellular space (Lkhider et al., 2004; Benes et al., 2008). The trigger for the proteolytic degradation of hemoglobin and the liberation of HBB(112–147) is an acidic pH which is a hallmark of inflammatory tissues and infections (Erra Díaz et al., 2018). A low pH not only activates the protease but also results in the dissociation of the hemoglobin subunits and the exposure of the otherwise buried proteolytic cleavage site (Huang et al., 2013). The second protease we found to release HBB(112–147) is Napsin A, a less explored aspartic protease present in the lung and kidney. While there is no description of Napsin A being expressed in placental tissue in literature, we detected Napsin A in placenta homogenate using specific antibodies. Our findings suggest that a local acidification at sites of infection or inflammation result in the rapid generation of HBB(112–147) that acts as broad-based antibacterial (and anti-HSV-2) agent.

The concentrations of HBB(112–147) that are required to suppress HSV-2 infection or bacterial growth are in the high μg/ml range. However, these seemingly high concentrations can be readily achieved *in vivo* because the hemoglobin precursor and the AMP-releasing protease are present almost everywhere in the human body. Hemoglobin is the second most abundant protein in humans with concentrations of 12–20 g in every 100 ml of human blood, and 50 g in spleen and bone marrow. 15 g of the tetrameric hemoglobin per 100 ml blood corresponds to 150 mg per 1 ml, and ∼75 mg of the β-hemoglobin subunits per ml blood. Complete proteolytic processing of hemoglobin (as it occurred in our *in vitro* digestions) would result in concentrations of ∼18 mg/ml of HBB(112–147). Even if only 10% of available hemoglobin is digested, this would still correspond to ∼1800 μg/ml, which is sufficient to block HSV-2 infection or bacterial growth entirely. These theoretical considerations are in line with experimental results obtained in previous studies (Liepke et al., 2003; Ständker et al., 2010) that revealed HBB(112–147) concentrations between 280 and 740 μg/g placenta tissue in different placenta samples, corresponding to an average concentration of 570 μg/g or ml. Thus, HBB(112–1476) may represent the AMP with the highest reported concentration in a human tissue and may play a key role in restricting transmission of viral and bacterial pathogens across the placenta and perhaps also other compartments.

## Data Availability Statement

The raw data supporting the conclusions of this article will be made available by the authors, without undue reservation, to any qualified researcher.

## Author Contributions

RG performed HSV-2, HIV-1, and ZIKV experiments, performed protease digestions, and edited the manuscript. RB analyzed the effect of HBB peptide on bacteria. FKr analyzed the effect of HBB peptide on HSV-1, HIV-1, and ZIKV. ER-B performed the initial screens and time of addition experiments. L-RO performed Napsin digestions. NP and LS generated the placenta libraries, purified or synthesized HBB(112–147). AR, LS, and SW were responsible for mass spec analysis. CC helped with HSV-2 assays. DS performed the evolutionary analysis. FKi supervised work and co-designed the study. BH isolated carbapenem-resistant PA. JG and TW did CD spectroscopy. YR-B and ES-G provided the structural analysis of the model of hemoglobin. W-GF provided a placenta library and purified HBB(112–147). AM and SSa performed and analyzed RUBV experiments. PW helped with TEM. JM and BS designed all experiments, supervised the study and wrote the manuscript. FG and SSt performed analysis of Napsin A expression in placental tissue.

## Conflict of Interest

ER-B, LS, W-GF, FK, and JM were inventors of patents claiming the use of HBB(112–147) as antiviral and antibacterial agent. W-GF was employed by Pharis Biotech. The remaining authors declare that the research was conducted in the absence of any commercial or financial relationships that could be construed as a potential conflict of interest.

## Footnotes

1 http://www.bioinfor.com/peaks-studio

2 www.uniprot.org

3 http://www.genome.jp/tools/clustalw/

4 http://www.ebi.ac.uk/thornton-srv/databases/cgi-bin/valdar/scorecons_server.pl

## References

[B1] Abrahams V. M. Bole-Aldo P. Kim Y. M. Straszewski-Chavez S. L. Chaiworapongsa T. Romero R. (2004). Divergent trophoblast responses to bacterial products mediated by TLRs. *J. Immunol.* 173 4286–4296. 10.4049/jimmunol.173.7.4286 15383557

[B2] Arnold F. Schnell J. Zirafi O. Stürzel C. Meier C. Weil T. (2012). Naturally occurring fragments from two distinct regions of the prostatic acid phosphatase form amyloidogenic enhancers of HIV infection. *J. Virol.* 86 1244–1249. 10.1128/JVI.06121-11 22090109 PMC3255800

[B3] Avgil M. Ornoy A. (2006). Herpes simplex virus and Epstein-Barr virus infections in pregnancy: consequences of neonatal or intrauterine infection. *Reprod. Toxicol.* 21 436–445. 10.1016/j.reprotox.2004.11.014 16580943

[B4] Barrett A. J. Cathepsin D. (1970). Purification of isoenzymes from human and chicken liver. *Biochem. J.* 117 601–607. 10.1042/bj1170601 5419752 PMC1178965

[B5] Benes P. Vetvicka V. Fusek M. (2008). Cathepsin D-Many functions of one aspartic protease. *Crit. Rev. Oncol. Hematol.* 68 12–28. 10.1016/j.critrevonc.2008.02.008 18396408 PMC2635020

[B6] Bensch K. W. Raida M. Mägert H. J. Schulz-Knappe P. Forssmann W. G. (1995). hBD-1: a novel β-defensin from human plasma. *FEBS Lett.* 368 331–335. 10.1016/0014-5793(95)00687-5 7628632

[B7] Berger J. R. Houff S. (2008). Neurological complications of herpes simplex virus type 2 infection. *Arch. Neurol.* 65 596–600. 10.1001/archneur.65.5.596 18474734

[B8] Bosso M. Ständker L. Kirchhoff F. Münch J. (2018). Exploiting the human peptidome for novel antimicrobial and anticancer agents. *Bioorg. Med. Chem.* 26 2719–2726. 10.1016/j.bmc.2017.10.038 29122440

[B9] Brown Z. A. Gardella C. Wald A. Morrow R. A. Corey L. (2005). Genital herpes complicating pregnancy. *Obstet. Gynecol.* 106 845–856. 10.1097/01.aog.0000180779.35572.3a 16199646

[B10] Brown Z. A. Wald A. Morrow R. A. Zeh J. Corey L. (2003). Effect of serologic status and cesarean delivery on transmission rates of herpes simplex virus from mother to infant. *J. Am. Med. Assoc.* 289 203–209. 12517231 10.1001/jama.289.2.203

[B11] Chouard T. (2011). Structural biology: breaking the protein rules. *Nature* 471 151–153. 10.1038/471151a 21390105

[B12] CPSP (2002). Neonatal herpes simplex virus infection: a devastating newborn pathogen. *Paediatr. Child Health* 7:19. 10.1093/pch/7.1.19 20046269 PMC2794524

[B13] Detheux M. Ständker L. Vakili J. Münch J. Forssmann U. Adermann K. (2000). Natural proteolytic processing of hemofiltrate CC chemokine 1 generates a potent CC chemokine receptor (CCR)1 and CCR5 agonist with anti-HIV properties. *J. Exp. Med.* 192 1501–1508. 10.1084/jem.192.10.1501 11085751 PMC2193185

[B14] Dick G. W. A. Kitchen S. F. Haddow A. J. (1952). Zika virus. I. Isolations and serological specificity. *Trans. R. Soc. Trop. Med. Hyg.* 46 509–520. 10.1016/0035-9203(52)90042-4 12995440

[B15] Diment S. Martin K. J. Stahl P. D. (1989). Cleavage of parathyroid hormone in macrophage endosomes illustrates a novel pathway for intracellular processing of proteins. *J. Biol. Chem.* 264 13403–13406. 2760027

[B16] Dolan A. Jamieson F. E. Cunningham C. Barnett B. C. McGeoch D. J. (1998). The genome sequence of herpes simplex virus type 2. *J. Virol.* 72 2010–2021. 10.1128/jvi.72.3.2010-2021.1998 9499055 PMC109494

[B17] Dürr U. H. N. Sudheendra U. S. Ramamoorthy A. (2006). LL-37, the only human member of the cathelicidin family of antimicrobial peptides. *Biochim. Biophys. Acta Biomembr.* 1758 1408–1425. 10.1016/j.bbamem.2006.03.030 16716248

[B18] Dyer A. P. Banfield B. W. Martindale D. Spannier D. M. Tufaro F. (1997). Dextran sulfate can act as an artificial receptor to mediate a type-specific herpes simplex virus infection via glycoprotein B. *J. Virol.* 71 191–198. 10.1128/jvi.71.1.191-198.1997 8985338 PMC191039

[B19] Dyson H. J. Wright P. E. (2005). Intrinsically unstructured proteins and their functions. *Nat. Rev. Mol. Cell Biol.* 6 197–208. 10.1038/nrm1589 15738986

[B20] Easterhoff D. Ontiveros F. Brooks L. R. Kim Y. Ross B. Silva J. N. (2013). Semen-derived enhancer of viral infection (SEVI) binds bacteria, enhances bacterial phagocytosis by macrophages, and can protect against vaginal infection by a sexually transmitted bacterial pathogen. *Antimicrob. Agents Chemother.* 57 2443–2450. 10.1128/AAC.02464-12 23507280 PMC3716189

[B21] Erra Díaz F. Dantas E. Geffner J. (2018). Unravelling the interplay between extracellular acidosis and immune cells. *Mediators Inflamm.* 2018:1218297. 10.1155/2018/1218297 PMC6332927 30692870

[B22] Finger-Jardim F. Teixeira L. O. de Oliveira G. R. Barral M. F. da Hora V. P. Gonçalves C. V. (2014). Herpes simplex virus: prevalence in placental tissue and incidence in neonatal cord blood samples. *J. Med. Virol.* 86 519–524. 10.1002/jmv.23817 24375504

[B23] Finsterbusch T. Wolbert A. Deitemeier I. Meyer K. Mosquera M. M. Mankertz A. (2009). Measles viruses of genotype H1 evade recognition by vaccine-induced neutralizing antibodies targeting the linear haemagglutinin noose epitope. *J. Gen. Virol.* 90 2739–2745. 10.1099/vir.0.013524-0 19625457

[B24] Flagg E. W. Weinstock H. (2011). Incidence of neonatal herpes simplex virus infections in the United States, 2006. *Pediatrics* 127 e1–e8. 10.1542/peds.2010-0134 21149432

[B25] Forssmann W.-G. The Y. H. Stoll M. Adermann K. Albrecht U. Tillmann H. C. (2010). Short-term monotherapy in HIV-infected patients with a virus entry inhibitor against the gp41 fusion peptide. *Sci. Transl. Med.* 2:63re3. 10.1126/scitranslmed.3001697 21178138

[B26] Hagemann J. B. Pfennigwerth N. Gatermann S. G. von Baum H. Essig A. (2018). KPC-2 carbapenemase-producing *Pseudomonas aeruginosa* reaching Germany. *J. Antimicrob. Chemother.* 73 1812–1814. 10.1093/jac/dky105 29590370

[B27] Herold B. C. Gerber S. I. Belval B. J. Siston A. M. Shulman N. (1996). Differences in the susceptibility of herpes simplex virus types 1 and 2 to modified heparin compounds suggest serotype differences in viral entry. *J. Virol.* 70 3461–3469. 10.1128/jvi.70.6.3461-3469.1996 8648678 PMC190219

[B28] Hertz C. J. Wu Q. Porter E. M. Zhang Y. J. Weismüller K. H. Godowski P. J. (2003). Activation of toll-like receptor 2 on human tracheobronchial epithelial cells induces the antimicrobial peptide human β defensin-2. *J. Immunol.* 171 6820–6826. 10.4049/jimmunol.171.12.6820 14662888

[B29] Huang Y. X. Wu Z. J. Huang B. T. Luo M. (2013). Pathway and mechanism of pH dependent human hemoglobin tetramer-dimer- monomer dissociations. *PLoS One* 8:e81708. 10.1371/journal.pone.0081708 24312337 PMC3842943

[B30] Hutton R. D. Ewert D. L. French G. R. (1973). Differentiation of types 1 and 2 herpes simplex virus by plaque inhibition with sulfated polyanions. *Proc. Soc. Exp. Biol. Med.* 142 27–29. 10.3181/00379727-142-36950 4345720

[B31] John H. Huynh K. D. Hedtmann C. Walden M. Schulz A. Anspach F. B. (2005). In vitro degradation of the antimicrobial human peptide HEM-gamma 130-146 in plasma analyzed by a validated quantitative LC-MS/MS procedure. *Anal. Biochem.* 341 173–186. 10.1016/j.ab.2005.03.025 15866542

[B32] Kacprzyk L. Rydengård V. Mörgelin M. Davoudi M. Pasupuleti M. Malmsten M. (2007). Antimicrobial activity of histidine-rich peptides is dependent on acidic conditions. *Biochim. Biophys. Acta* 1768 2667–2680. 10.1016/j.bbamem.2007.06.020 17655823

[B33] Khatami M. H. Bromberek M. Saika-Voivod I. Booth V. (2014). Molecular dynamics simulations of histidine-containing cod antimicrobial peptide paralogs in self-assembled bilayers. *Biochim. Biophys. Acta* 1838 2778–2787. 10.1016/j.bbamem.2014.07.013 25058381

[B34] Kimberlin D. W. (2007). Herpes simplex virus infections of the newborn. *Semin. Perinatol.* 31 19–25. 10.1053/j.semperi.2007.01.003 17317423

[B35] Koi H. Zhang J. Makrigiannakis A. Getsios S. Maccalman C. D. Strauss J. F. (2002). Syncytiotrophoblast is a barrier to maternal-fetal transmission of herpes simplex virus1. *Biol. Reprod.* 67 1572–1579. 10.1095/biolreprod.102.004325 12390890

[B36] Krause A. Neitz S. Mägert H. J. Schulz A. Forssmann W. G. Schulz-Knappe P. (2000). LEAP-1, a novel highly disulfide-bonded human peptide, exhibits antimicrobial activity. *FEBS Lett.* 480 147–150. 10.1016/s0014-5793(00)01920-7 11034317

[B37] Krause A. Sillard R. Kleemeier B. Klüver E. Maronde E. Conejo-García J. R. (2003). Isolation and biochemical characterization of LEAP-2, a novel blood peptide expressed in the liver. *Protein Sci.* 12 143–152. 10.1110/ps.0213603 12493837 PMC2312392

[B38] Krüger F. Kumar V. Monge P. Conzelmann C. Smith N. Gothelf K. V. (2019). Nucleic acids as a nature-inspired scaffold for macromolecular prodrugs of nucleoside analogues. *Adv. Sci.* 6:1802095. 10.1002/advs.201802095 30937274 PMC6425433

[B39] Langeland N. Holmsen H. Lillehaug J. R. Haarr L. (1987). Evidence that neomycin inhibits binding of herpes simplex virus type 1 to the cellular receptor. *J. Virol.* 61 3388–3393. 10.1128/jvi.61.11.3388-3393.1987 2822948 PMC255933

[B40] Langeland N. Moore L. J. Holmsen H. Haarr L. (1988). Interaction of polylysine with the cellular receptor for herpes simplex virus type 1. *J. Gen. Virol.* 69(Pt 6), 1137–1145. 10.1099/0022-1317-69-6-1137 2838567

[B41] Liepke C. Baxmann S. Heine C. Breithaupt N. Ständker L. Forssmann W. G. (2003). Human hemoglobin-derived peptides exhibit antimicrobial activity: a class of host defense peptides. *J. Chromatogr. B Analyt. Technol. Biomed. Life Sci.* 791 345–356. 10.1016/s1570-0232(03)00245-9 12798194

[B42] Liepke C. Zucht H. D. Forssmann W. G. Ständker L. (2001). Purification of novel peptide antibiotics from human milk. *J. Chromatogr. B* 752 369–377. 10.1016/s0378-4347(00)00516-8 11270874

[B43] Lkhider M. Castino R. Bouguyon E. Isidoro C. Ollivier-Bousquet M. (2004). Cathepsin D released by lactating rat mammary epithelial cells is involved in prolactin cleavage under physiological conditions. *J. Cell Sci.* 117 5155–5164. 10.1242/jcs.01396 15456852

[B44] Looker K. J. Magaret A. S. Turner K. M. Vickerman P. Gottlieb S. L. Newman L. M. (2015). Global estimates of prevalent and incident herpes simplex virus type 2 infections in 2012. *PLoS One* 10:e114989. 10.1371/journal.pone.0114989 25608026 PMC4301914

[B45] Mahnert N. Roberts S. W. Laibl V. R. Sheffield J. S. Wendel G. D. (2007). The incidence of neonatal herpes infection. *Am. J. Obstet. Gynecol.* 196 E55–E56. 17466681 10.1016/j.ajog.2006.10.911

[B46] Malhotra S. Hayes D. Wozniak D. J. (2019). Cystic Fibrosis and *Pseudomonas aeruginosa*: the host-microbe interface. *Clin. Microbiol. Rev.* 32:e00138-18. 10.1128/CMR.00138-18 PMC6589863 31142499

[B47] Malik E. Dennison S. R. Harris F. Phoenix D. (2016). A. pH dependent antimicrobial peptides and proteins, their mechanisms of action and potential as therapeutic agents. *Pharmaceuticals* 9:67. 10.3390/ph9040067 27809281 PMC5198042

[B48] Malkin J.-E. (2004). Epidemiology of genital herpes simplex virus infection in developed countries. *Herpes* 11(Suppl. 1), 2A–23A. 15115626

[B49] Mirny L. A. Shakhnovich E. I. (1999). Universally conserved positions in protein folds: reading evolutionary signals about stability, folding kinetics and function. 291 177–196. 10.1006/jmbi.1999.2911 10438614

[B50] Mohr K. B. Zirafi O. Hennies M. Wiese S. Kirchhoff F. Münch J. (2015). Sandwich enzyme-linked immunosorbent assay for the quantification of human serum albumin fragment 408–423 in bodily fluids. *Anal. Biochem.* 476 29–35. 10.1016/j.ab.2015.01.023 25660532

[B51] Mor G. Kwon J. Y. (2015). Trophoblast-microbiome interaction: a new paradigm on immune regulation. *Am. J. Obstet. Gynecol.* 213 S131–S137. 10.1016/j.ajog.2015.06.039 26428492 PMC6800181

[B52] Müller J. A. Harms M. Krüger F. Groß R. Joas S. Hayn M. (2018). Semen inhibits Zika virus infection of cells and tissues from the anogenital region. *Nat. Commun.* 9:2207. 10.1038/s41467-018-04442-y 29880824 PMC5992203

[B53] Münch J. Rücker E. Ständker L. Adermann K. Goffinet C. Schindler M. (2007a). Semen-derived amyloid fibrils drastically enhance HIV infection. *Cell* 131 1059–1071. 10.1016/j.cell.2007.10.014 18083097

[B54] Münch J. Ständker L. Adermann K. Schulz A. Schindler M. Chinnadurai R. (2007b). Discovery and optimization of a natural HIV-1 entry inhibitor targeting the gp41 fusion peptide. *Cell* 129 263–275. 10.1016/j.cell.2007.02.042 17448989

[B55] Münch J. Ständker L. Forssmann W. G. Kirchhoff F. (2014). Discovery of modulators of HIV-1 infection from the human peptidome. *Nat. Rev. Microbiol.* 12 715–722. 10.1038/nrmicro3312 25110191 PMC7097597

[B56] Münch J. Ständker L. Pöhlmann S. Baribaud F. Papkalla A. Rosorius O. (2002). Hemofiltrate CC chemokine 1[9-74] causes effective internalization of CCR5 and is a potent inhibitor of R5-tropic human immunodeficiency virus type 1 strains in primary T cells and macrophages. *Antimicrob. Agents Chemother.* 46 982–990. 10.1128/aac.46.4.982-990.2002 11897579 PMC127102

[B57] Papagianni M. (2003). Ribosomally synthesized peptides with antimicrobial properties: biosynthesis, structure, function, and applications. *Biotechnol. Adv.* 21 465–499. 10.1016/s0734-9750(03)00077-6 14499150

[B58] Papkalla A. Münch J. Otto C. Kirchhoff F. (2002). Nef enhances human immunodeficiency virus type 1 infectivity and replication independently of viral coreceptor tropism. *J. Virol.* 76 8455–8459. 10.1128/jvi.76.16.8455-8459.2002 12134048 PMC155138

[B59] Pendleton J. N. Gorman S. P. Gilmore B. F. (2013). Clinical relevance of the ESKAPE pathogens. *Expert Rev. Anti Infect. Ther.* 11 297–308. 10.1586/eri.13.12 23458769

[B60] Piret J. Boivin G. (2011). Resistance of herpes simplex viruses to nucleoside analogues: mechanisms, prevalence, and management. *Antimicrob. Agents Chemother.* 55 459–472. 10.1128/AAC.00615-10 21078929 PMC3028810

[B61] Proffitt M. R. Schindler S. A. (1995). Rapid detection of HSV with an enzyme-linked virus inducible system (ELVIS) employing a genetically modified cell line. *Clin. Diagn. Virol.* 4 175–182. 10.1016/0928-0197(95)00011-v 15566838

[B62] Pulido D. Nogús M. V. Boix E. Torrent M. (2012). Lipopolysaccharide neutralization by antimicrobial peptides: a gambit in the innate host defense strategy. *J. Innate Immun.* 4 327–336. 10.1159/000336713 22441679 PMC6741597

[B63] Robbins J. R. Bakardjiev A. I. (2012). Pathogens and the placental fortress. *Curr. Opin. Microbiol.* 15 36–43. 10.1016/j.mib.2011.11.006 22169833 PMC3265690

[B64] Roberts S. (2009). Herpes simplex virus: incidence of neonatal herpes simplex virus, maternal screening, management during pregnancy, and HIV. *Curr. Opin. Obstet. Gynecol.* 21 124–130. 10.1097/gco.0b013e3283294840 19262380

[B65] Röcker A. Roan N. R. Yadav J. K. Fändrich M. Münch J. (2018). Structure, function and antagonism of semen amyloids. *Chem. Commun.* 54 7557–7569. 10.1039/c8cc01491d 29873340 PMC6033663

[B66] Schandock F. Riber C. F. Röcker A. Müller J. A. Harms M. Gajda P. (2017). Macromolecular antiviral agents against Zika, Ebola, SARS, and other pathogenic viruses. *Adv. Healthc. Mater.* 6:1700748. 10.1002/adhm.201700748 28945945 PMC7161897

[B67] Scoular A. (2002). Using the evidence base on genital herpes: optimising the use of diagnostic tests and information provision. *Sex Transm. Infect.* 78 160–165. 10.1136/sti.78.3.160 12238644 PMC1744455

[B68] Shieh M. T. WuDunn D. Montgomery R. I. Esko J. D. Spear P. G. (1992). Cell surface receptors for herpes simplex virus are heparan sulfate proteoglycans. *J. Cell Biol.* 116 1273–1281. 10.1083/jcb.116.5.1273 1310996 PMC2289355

[B69] Sitaram N. Nagaraj R. (2002). Host-defense antimicrobial peptides: importance of structure for activity. *Curr. Pharm. Des.* 8 727–742. 10.2174/1381612023395358 11945168

[B70] Smith J. S. Robinson N. J. (2002). Age-specific prevalence of infection with herpes simplex virus types 2 and 1: a global review. *J. Infect. Dis.* 186(Suppl. 1), S3–S28. 12353183 10.1086/343739

[B71] Ständker L. Zachgo V. Hillemanns P. Rösinger M. Forssmann W. G. Hass R. (2010). Quantitative enzyme-linked immunosorbent assay determination of an abundant hemoglobin-derived anti-infective peptide in human placenta. *Anal. Biochem.* 401 53–60. 10.1016/j.ab.2010.02.019 20188690

[B72] Sugase K. Dyson H. J. Wright P. E. (2007). Mechanism of coupled folding and binding of an intrinsically disordered protein. *Nature* 447 1021–1025. 10.1038/nature05858 17522630

[B73] Trybala E. Liljeqvist J. A. Svennerholm B. Bergström T. (2000). Herpes simplex virus types 1 and 2 differ in their interaction with heparan sulfate. *J. Virol.* 74 9106–9114. 10.1128/jvi.74.19.9106-9114.2000 10982357 PMC102109

[B74] Weiss H. (2004). Epidemiology of herpes simplex virus type 2 infection in the developing world. *Herpes* 11(Suppl. 1), 24A–35A. 15115627

[B75] Wright P. E. Dyson H. J. (2015). Intrinsically disordered proteins in cellular signaling and regulation HHS public access. *Nat. Rev. Mol. Cell Biol.* 16 18–29. 25531225 10.1038/nrm3920 PMC4405151

[B76] Zanluca C. De Noronha L. Duarte C. N. Santos D. (2018). Maternal-fetal transmission of the zika virus: an intriguing interplay. *Tissue Barriers* 6:e1402143. 10.1080/21688370.2017.1402143 29370577 PMC5823548

[B77] Zhang J. Xin L. Shan B. Chen W. Xie M. Yuen D. (2012). PEAKS DB: de novo sequencing assisted database search for sensitive and accurate peptide identification. *Mol. Cell. Proteomics* 11:M111.010587. 10.1074/mcp.M111.010587 22186715 PMC3322562

[B78] Zirafi O. Kim K. A. Ständker L. Mohr K. B. Sauter D. Heigele A. (2015). Discovery and characterization of an endogenous CXCR4 antagonist. *Cell Rep.* 11 737–747. 10.1016/j.celrep.2015.03.061 25921529

