# Supplementary material for: A Placenta Derived C-Terminal Fragment of β-Hemoglobin With Combined Antibacterial and Antiviral Activity
Source: Front Microbiol. 2020 Apr 6;11:508. doi: 10.3389/fmicb.2020.00508 (PMC7153485; doi:10.3389/fmicb.2020.00508)
Supplement: Supplementary file 1 [file Data_Sheet_1.docx]

Supplementary Material

A placenta derived C-terminal fragment of β-hemoglobin with

combined antibacterial and antiviral activity

Rüdiger Groß^1*^, Richard Bauer^2*^, Franziska Krüger^1*^, Elke Rücker-Braun^3*^, Lia-Raluca Olari^1^, Ludger Ständker^4^, Nico Preising^4^, Armando A. Rodríguez^4,11^, Carina Conzelmann^1^, Fabian Gerbl^2^, Daniel Sauter^1^, Frank Kirchhoff^1^, Benjamin Hagemann^2^, Jasmina Gačanin^5,6^, Tanja Weil^5,6^, Yasser B. Ruiz-Blanco^7^, Elsa Sanchez-Garcia^7^, Wolf-Georg Forssmann^8^, Annette Mankertz^9^, Sabine Santibanez^9^, Steffen Stenger^2^, Paul Walther^10^, Sebastian Wiese^11^, Barbara Spellerberg^2§^, Jan Münch^1,4§^

# Supplementary Figures


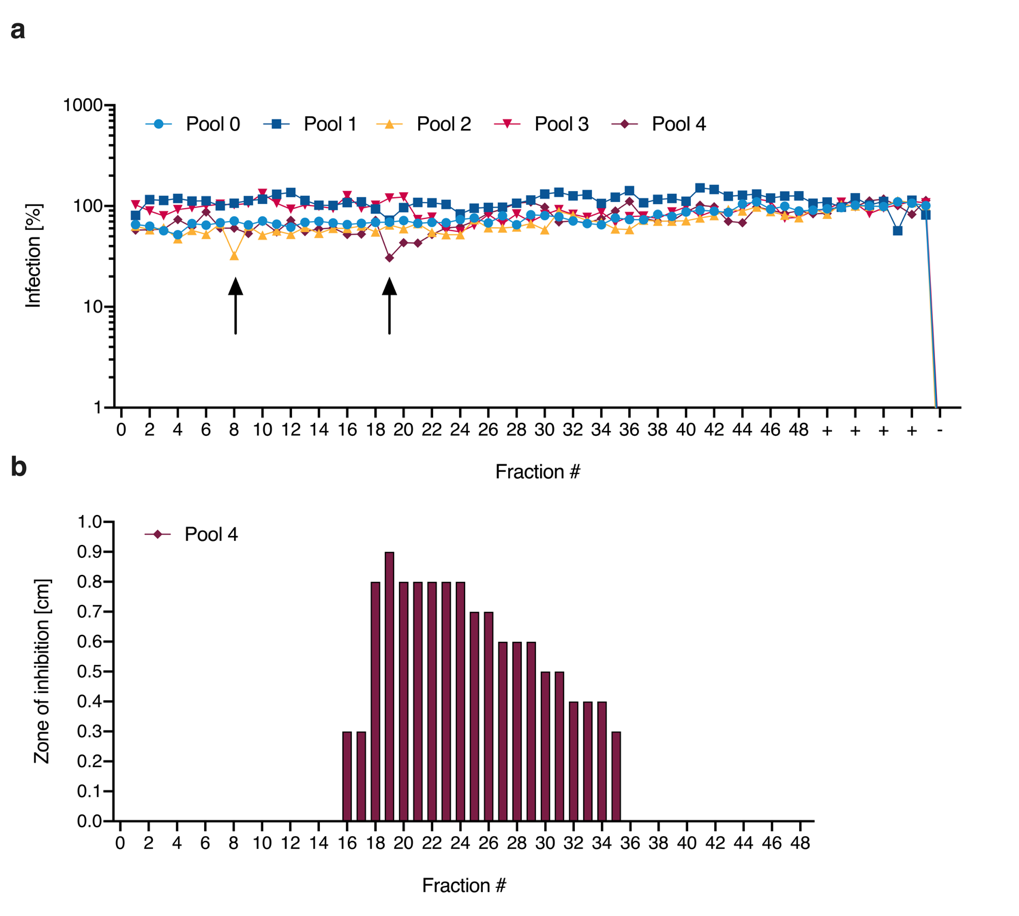


**Supplementary Figure 1. Effect of fractions of a placenta derived peptide library on HSV-2 infection and *P. aeruginosa* growth.** a) Fractions 1-48 of pH pools 0-4 of a placenta peptide library were dissolved in PBS and added to ELVIS^TM^ cells, which were subsequently infected with a clinical HSV-2 isolate. Infection rates were determined 2 days later by quantifying ß-galactosidase activities in cellular lysates. Values shown are relative those obtained in the presence of buffer only (+). -, uninfected. Experiment was performed in triplicates, SDs are omitted for clarity. Fractions reducing HSV-2 infection by more than 50 % are encircled. b) The placental library was tested for antimicrobial activity against *Pseudomonas aeruginosa* in a radial diffusion assay. Inhibition zones are depicted in cm. Fraction 19 of eluate pool 4-6 showing highest antimicrobial activity contains HBB(112-147).


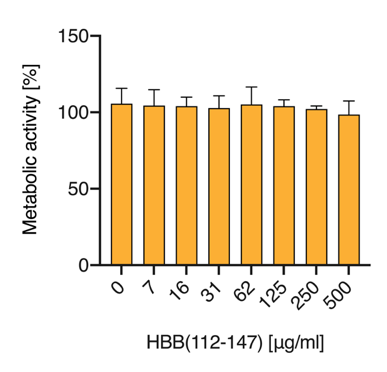


**Supplementary Figure 2. Synthetic HBB(112-147) is not cytotoxic.** ELVIS cells were incubated with indicated concentrations of HBB(112-147) for 3 days and metabolic activity was determined by MTT assay. Shown are average values derived from triplicates ± SD.


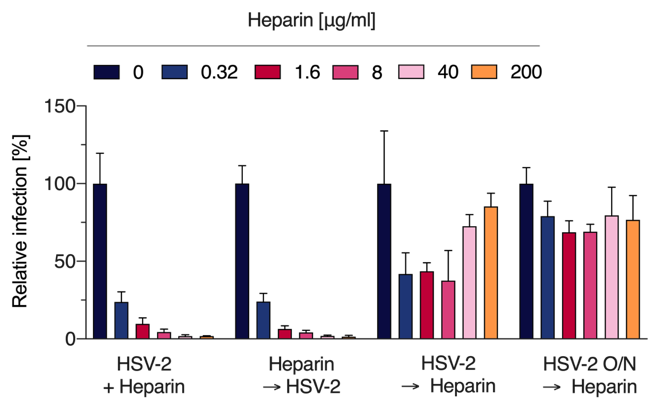


**Supplementary Figure 3. Inhibition of HSV-2 infection by heparin in time-of-addition experiments.** In analogy to time-of-addition experiments performed with HBB(112-147), Heparin was added at indicated concentrations either directly to cells prior to infection, pre-incubated for 30 min on cells at 37°C, added 2h post-infection (with removal of virus and addition of fresh medium) or added after overnight infection (16 hpi). Infection was evaluted by quantifying β-galactosidase activites in cellular lysates 24 hpi. Shown are average values derived from one experiment in triplicates ± SD

**Supplementary Figure 4. HIV-1 and ZIKV are not affected by HBB(112-147) in virion-treatment mode.** HIV-1 virions (R5-tropic NL4-3) or ZIKV (MR766) were exposed up to 1 mg/ml HBB(112-147) or CLR01 (as a positive control) for 30 min at 37°C before being added to TZM-bl cells (for HIV-1) or VeroE6 cells (for ZIKV) resulting in 5-fold dilution of compounds. Infection was evaluated 3 dpi by measuring ß-galactosidase activities in cellular lysates (HIV-1) or 2 dpi by an in-cell ELISA that detected the viral E protein (ZIKV).

**
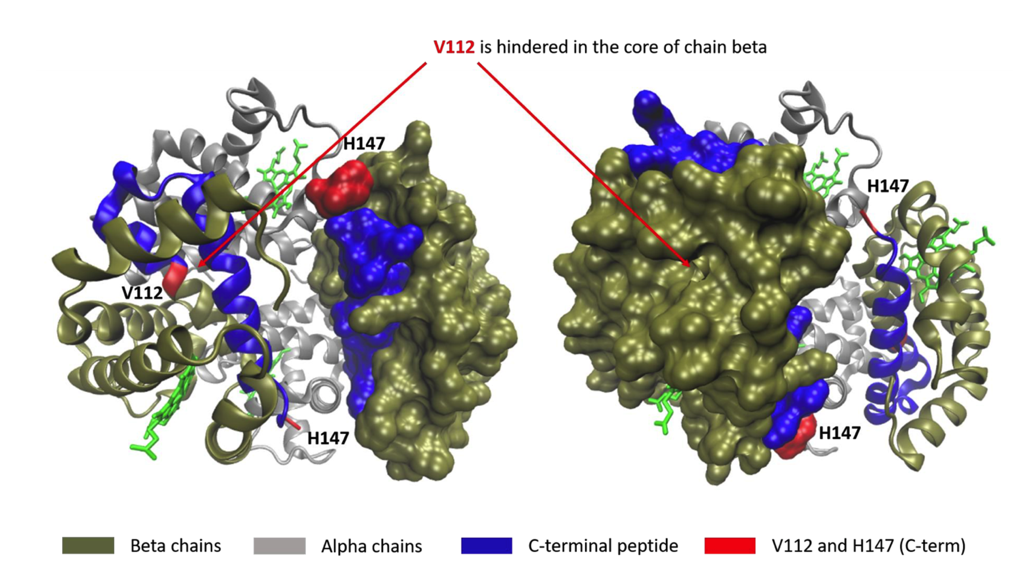
**

**Supplementary Figure 5. The putative protease cleavage site at position V112 is not accessible for proteases in the tetrameric state of hemoglobin.** The tetramer of hemoglobin is shown (PDB code: 2DN2) with the complete C-terminal HBB(112-147) peptides highlighted in blue and V112 and H147 residues highlighted in red, the heme groups are shown in green. β and α units of the tetramer are shown in olive green and grey, respectively. The representation of one β monomeric unit as surface further evidences that V112 is not solvent-accessible.

**
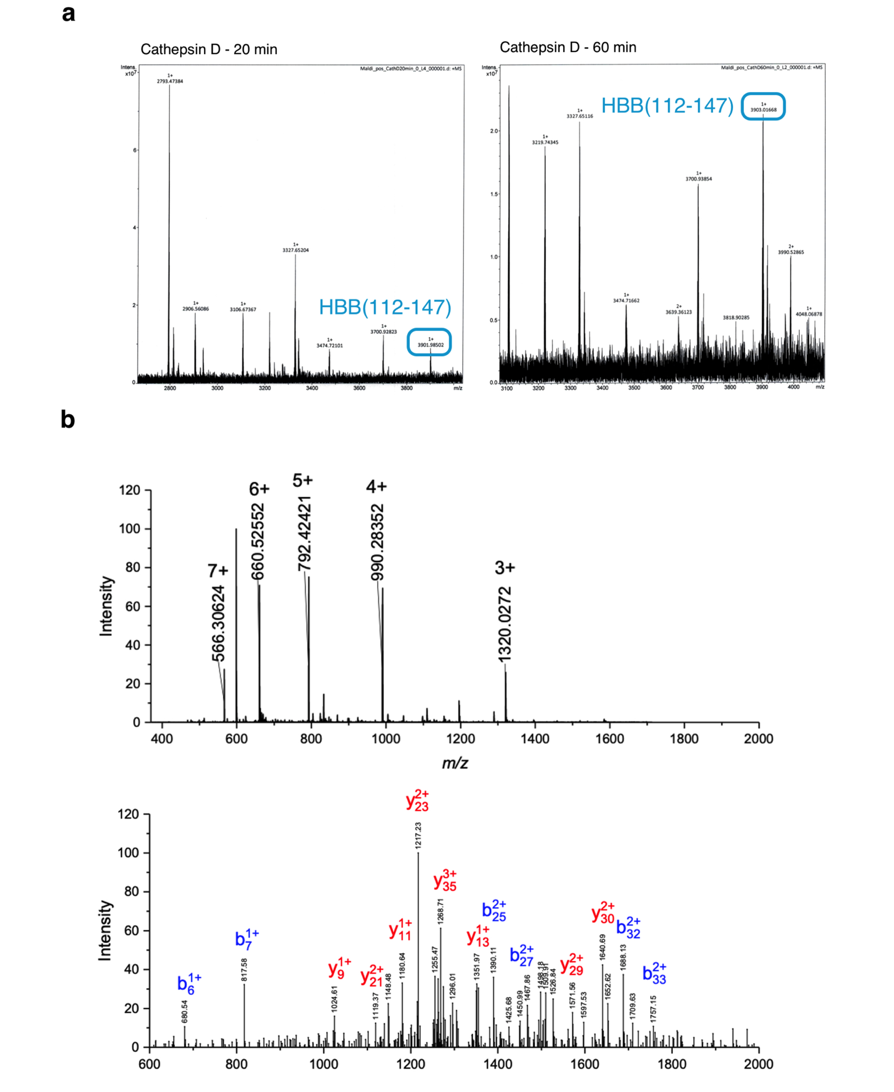
**

**Supplementary Figure 6. Detection of HBB(112-147) in proteolytic digestion by mass spectrometry.** (a) Detection of HBB(112-147) in Cathepsin D-digested hemoglobin by MALDI-TOF-MS. (b) Detection of HBB(112-147) in Napsin A-digested hemoglobin by LC-ESI-MS after carbamidomethylation. (above) MS-spectrum showing intact HBB(112-147) ions at indicated charge states. (below) Corresponding fragmentation spectrum of the HBB(112-147)^3+^ ion. Dominant fragments are labelled accordingly.

**
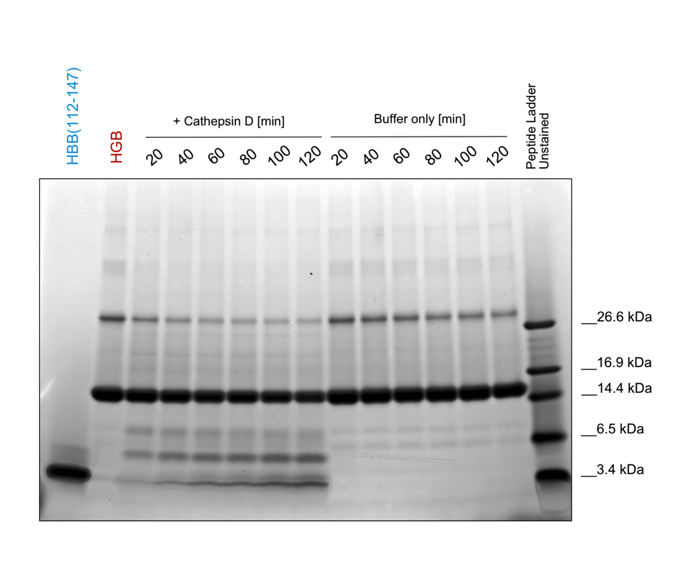
**


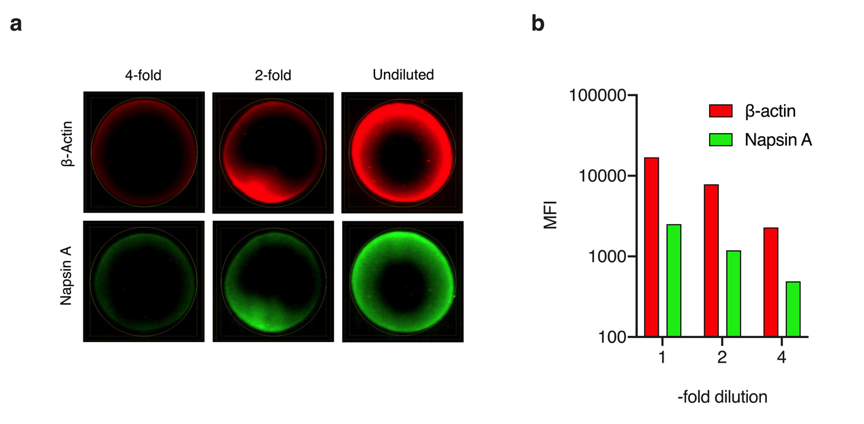
**Supplementary Figure 7. Additional information on generation of HBB(112-147) by proteolytic digestion:** Digestion kinetics of human hemoglobin with Cathepsin D (1:100 molar ratio) or buffer only (0.2M citrate, pH 5.0) over a time frame of 20 to 120 min.

**Supplementary Figure 8. Detection of Napsin A in placenta homogenate by dot blot.** Placental tissue was homogenized as described^30^. Before dot-blot, the extract was buffer-exchanged to PBS by ultrafiltration (3 kDa MWCO) and 3 washing steps (20 ml each). (a) Fluorescence images and (b) MFI quantification of β-actin (as a control, red) and Napsin A (green).

**
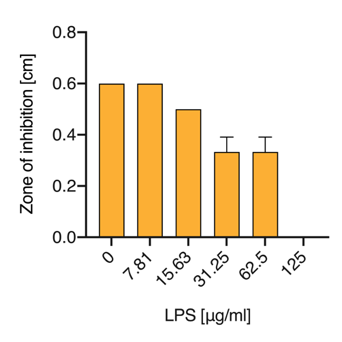
Supplementary Figure 9. Evolutionary conservation of HBB112-147 among vertebrates.** β-hemoglobin protein sequences of the indicated vertebrate species were aligned using Clustal W (bottom right) and residue conservation was determined using the Scorecons Server (top). The conservation scores of residues within (red) and outside (blue) HBB112-147 were directly compared (bottom left).

**Supplementary Figure 10.** **Inhibitory effect of LPS on the activity of HBB(112-147) against P. aeruginosa BSU856.** The effect of preincubation of 62.5 µg/ml HBB(112-147) with indicated concentrations of LPS of *P. aeruginosa* was determined in a radial diffusion assay. The mean values and standard deviations of three independent experiments are indicated.
